# Sildenafil improves hippocampal brain injuries and restores neuronal development after neonatal hypoxia–ischemia in male rat pups

**DOI:** 10.1038/s41598-021-01097-6

**Published:** 2021-11-11

**Authors:** Armin Yazdani, Belal Howidi, Meng Zhu Shi, Nicol Tugarinov, Zehra Khoja, Pia Wintermark

**Affiliations:** 1 Research Institute of the McGill University Health Centre, Montreal, Canada; 2 Division of Newborn Medicine, Department of Pediatrics, Montreal Children’s Hospital, 1001 boul. Décarie, Site Glen Block E, EM0.3244, Montreal, QC H4A 3J1 Canada

**Keywords:** Neurogenesis, Neurology, Neurological disorders, Neuroscience, Myelin biology and repair, Neurogenesis, Regeneration and repair in the nervous system

## Abstract

The hippocampus is a fundamental structure of the brain that plays an important role in neurodevelopment and is very sensitive to hypoxia–ischemia (HI). The purpose of this study was to investigate the effects of sildenafil on neonatal hippocampal brain injuries resulting from HI, and on neuronal development in this context. HI was induced in male Long–Evans rat pups at postnatal day 10 (P10) by a left common carotid ligation followed by a 2-h exposure to 8% oxygen. Rat pups were randomized to vehicle or sildenafil given orally twice daily for 7 days starting 12 h after HI. Hematoxylin and eosin staining was performed at P30 to measure the surface of the hippocampus; immunohistochemistry was performed to stain neurons, oligodendrocytes, and glial cells in the hippocampus. Western blots of the hippocampus were performed at P12, P17, and P30 to study the expression of neuronal markers and mTOR pathway. HI caused significant hippocampal atrophy and a significant reduction of the number of mature neurons, and induced reactive astrocytosis and microgliosis in the hippocampus. HI increased apoptosis and caused significant dysregulation of the normal neuronal development program. Treatment with sildenafil preserved the gross morphology of the hippocampus, reverted the number of mature neurons to levels comparable to sham rats, significantly increased both the immature and mature oligodendrocytes, and significantly reduced the number of microglia and astrocytes. Sildenafil also decreased apoptosis and reestablished the normal progression of post-natal neuronal development. The PI3K/Akt/mTOR pathway, whose activity was decreased after HI in the hippocampus, and restored after sildenafil treatment, may be involved. Sildenafil may have both neuroprotective and neurorestorative properties in the neonatal hippocampus following HI.

## Introduction

Neonatal hypoxia–ischemia (HI) results in extensive brain damage. Targeting the endogenous *neurorestorative* capacity of the neonatal brain following HI may improve the long-term neurological outcome of the affected neonates. It is known that the postnatal human brain can generate new neurons and oligodendrocytes through the proliferation of neural stem cells found in the subventricular zone of the lateral ventricles and the subgranular zone (SGZ) of the dentate gyrus (DG) of the hippocampus^1–3^. Activation of endogenous repair mechanisms such as neurogenesis and oligodendrogenesis may represent an innovative therapeutic target^1–4^. The hippocampus is an important site of endogenous repair processes after HI, since new neurons continue to be generated there in the postnatal period due to the existence of neural stem cells in the hippocampal subgranular zone (SGZ). The mammalian target of rapamycin (mTOR), a serine/threonine kinase, is one of the identified pathways involved in the promotion of the transition of NSCs into mature neurons^11,40^. Its dysregulation has been associated with an imbalance of neural progenitors that leads to severe neurologic deficits such as autism, epilepsy, and neurodegenerative disorders^47^. The developing hippocampus is important for learning and memory^5^. The hippocampus is a highly active brain region metabolically, with a high demand for oxygen^6^, and so it is an important target for hypoxic-ischemic insults^7^. Sildenafil, a selective inhibitor of phosphodiesterase type-5 (PDE5), has been demonstrated to be beneficial in repairing cortical and retinal injuries resulting from HI at a term-equivalent age by increasing the neuronal count^8,9^. Previous studies also have demonstrated that sildenafil ameliorates cortical brain injuries in both older and younger rats models of HI^4,10–12^. However, the effects of this treatment on the neonatal hippocampus have not yet been assessed. In addition, the effects of sildenafil on the neuronal development of the injured neonatal brain have not been studied previously.

Thus, the objective of this study was to investigate the effects of sildenafil on neonatal hippocampal brain injuries after HI at term-equivalent age and also explore the role of this treatment on neurons and on the mTOR pathway in this context. We hypothesize that sildenafil may have a neuroprotective role and a neurorestorative role that can ameliorate hippocampal brain injuries.

## Results

### Sildenafil treatment after HI decreased hippocampal injury

**Figure 1:**
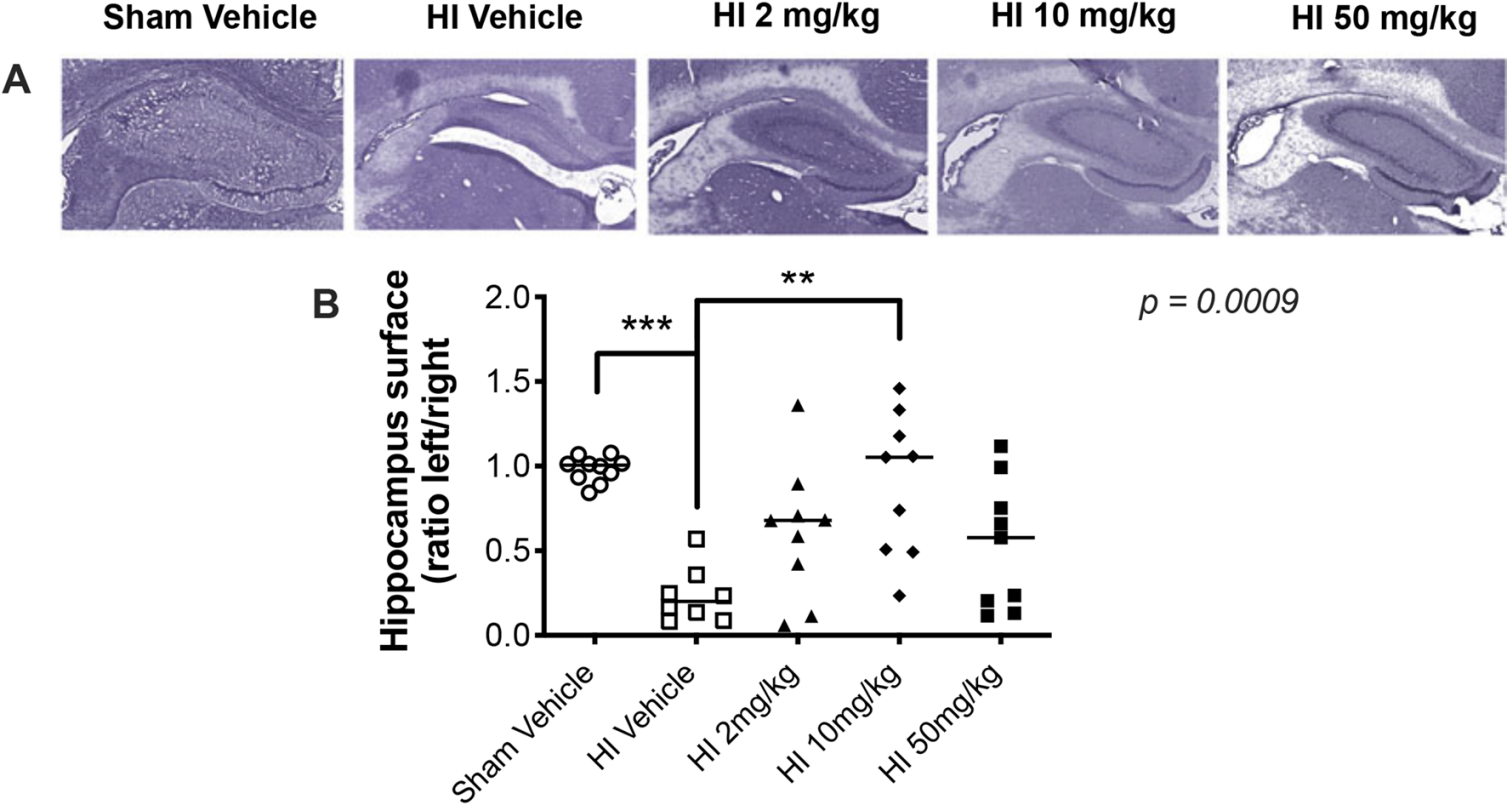
Hematoxylin and eosin-stained coronal brain sections in sham and HI rats treated with vehicle or sildenafil. (**A**) Representative hematoxylin and eosin-stained coronal brain sections showing the ipsilateral hippocampus. (**B**) Ipsilateral/contralateral hippocampus surface ratio (median with individual data points representation). Significance: *p* value from Kruskal–Wallis test, with Dunn’s post-hoc comparison tests: **p < 0.01, ***p < 0.001. Number of animals: n = 10 sham rats treated with vehicle (sham vehicle), 8 HI rats treated with vehicle (HI vehicle), 9 HI rats treated with a low-dose (2 mg/kg) of sildenafil (HI 2 mg/kg), 9 HI rats treated with a medium-dose (10 mg/kg) of sildenafil (HI 10 mg/kg) and 9 HI rats treated with a high-dose (10 mg/kg) of sildenafil (HI 50 mg/kg).

HI significantly reduced the size of the ipsilateral hippocampus at P30, compared to the contralateral hippocampus (Fig. 1A). The ratio between the ipsilateral and contralateral hippocampi surfaces was significantly reduced in the HI rat pups treated with vehicle (*p* < 0.001), compared to the sham-operated rats treated with vehicle (Fig. 1B). All doses of the sildenafil treatment demonstrated improvements in the gross anatomical structure of the ipsilateral hippocampus (Fig. 1A). All the sildenafil doses increased the ratios between the ipsilateral and contralateral hippocampi surfaces to levels that were not more significantly different than sham rats.

### Sildenafil treatment after HI preserved the neuronal count in the hippocampus

**Figure 2:**
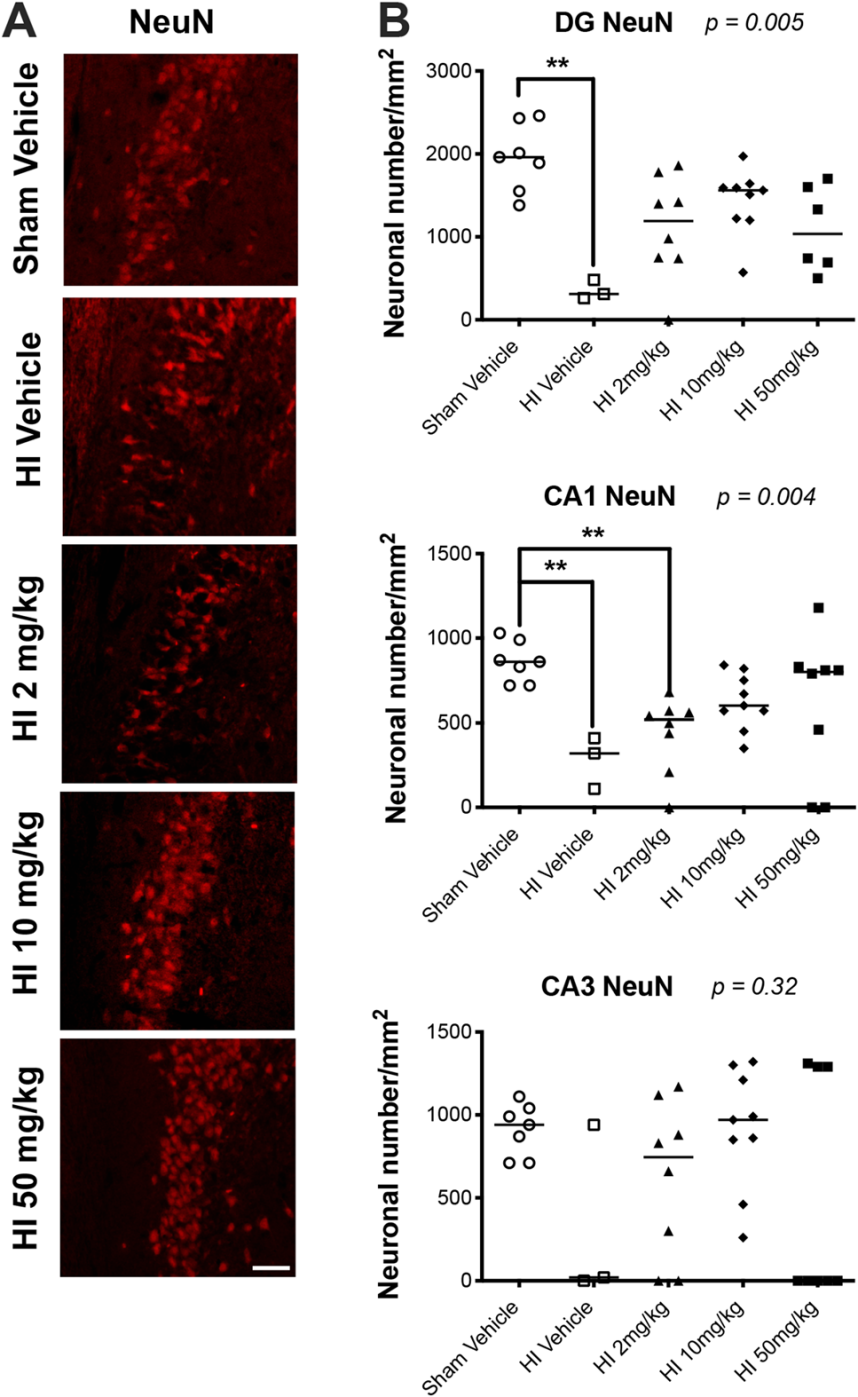
Immunostaining for mature neurons in sham and HI rats treated with vehicle or sildenafil. Cells were counted in three regions of the hippocampus: i.e., dentate gyrus (DG), CA1, and CA3 regions of the ipsilateral hippocampus. (**A**) Representative immunofluorescent micrographs of the mature neurons labeled with NeuN in the ipsilateral DG region of the hippocampus. (**B**) Mature neuronal cells labeled with NeuN (median with individual data points representation) in DG, CA1 and CA3. Significance: *p* value from Kruskal–Wallis test, with Dunn’s post-hoc comparison tests: **p < 0.01. Number of animals: n = 7 sham vehicle, 3 HI vehicle, 8 HI 2 mg/kg, 9 HI 10 mg/kg and 8 HI 50 mg/kg.

HI resulted in a significant decrease in the number of mature (NeuN+) neurons at P30 in the DG and CA1 regions of the ipsilateral hippocampus (Fig. 2A,B) (*p* < 0.01), compared to sham rats. The number of mature neurons also tended to decrease in the CA3 region of the ipsilateral hippocampus of HI rats treated with vehicle, but this number did not reach statistical significance, when compared to sham rats.

All doses of sildenafil reverted the number of mature neurons in the DG to levels no longer significantly different compared to sham rats (Fig. 2A,B). In addition, the medium-dose (10 mg/kg) and high-dose (50 mg/kg) of sildenafil reverted the number of mature neurons in the CA1 region to levels no longer significantly different compared to sham rats.

### Sildenafil treatment after HI decreased apoptosis and progressively restored the normal neuronal development program in the hippocampus

**Figure 3:**
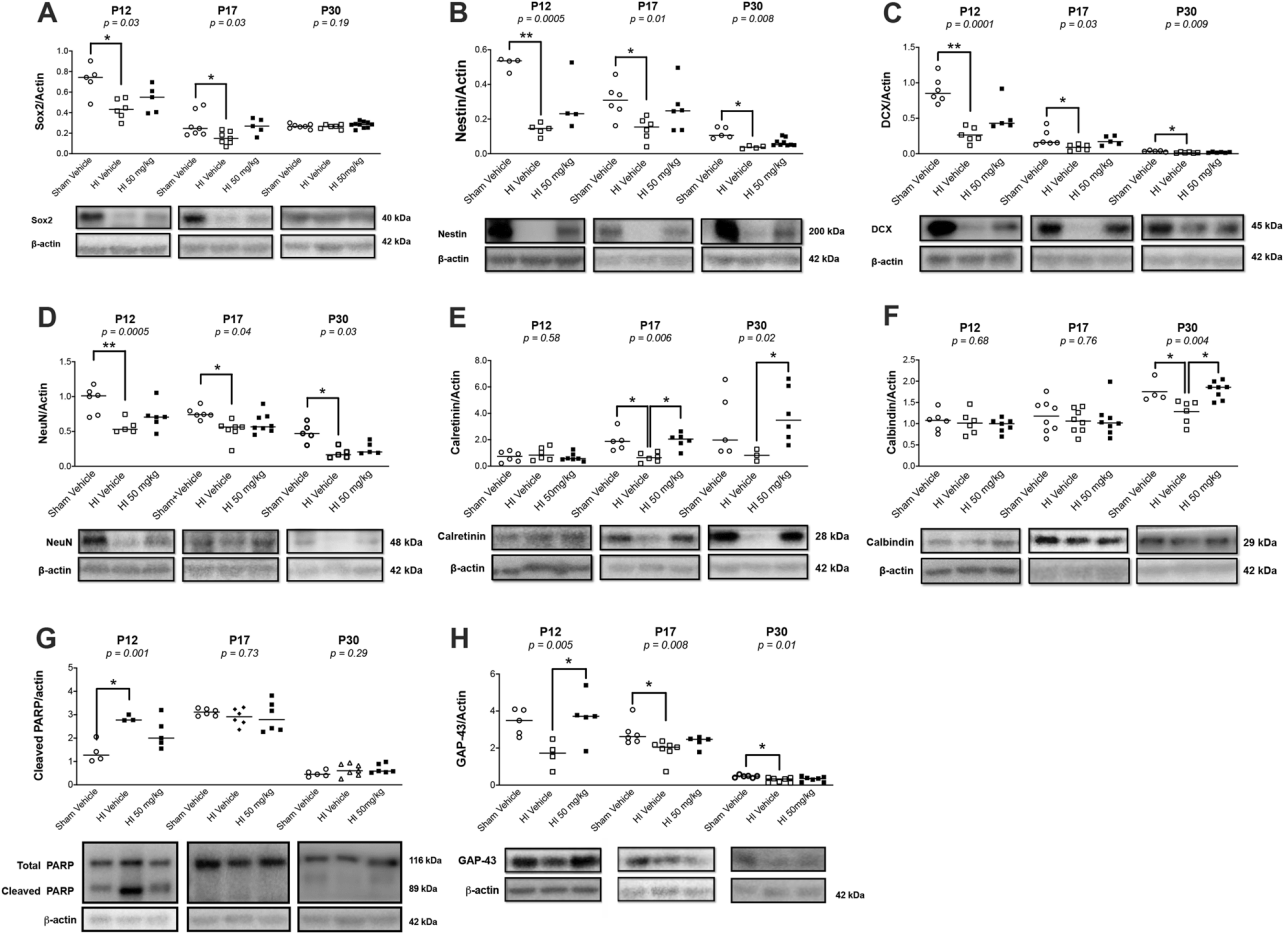
Western blotting for markers of early/late neuronal development, apoptosis, and axonal regeneration of the ipsilateral hippocampus in sham and HI rat pups treated with vehicle or sildenafil. Median with individual data points representation, with cropped representative western blots. Full-length blots are presented in Supplementary Material. (**A**) Sox2. (**B**) Nestin. (**C**) Doublecortin (DCX). (**D**) NeuN. (**E**) Calretinin. (**F**) Calbindin. (**G**) Cleaved PARP. (**H**) GAP-43. Significance: *p* value from Kruskal–Wallis test, with Dunn’s post-hoc comparison tests: *p < 0.05, **p < 0.01. Number of animals: n = 6, 8 and 7 sham vehicle, 6, 8 and 7 HI vehicle, and 7, 8 and 10 HI 50 mg/kg, respectively at P12, P17 and P30.

After HI, there was a significant reduction in the expression of both early and late neuronal development markers in the ipsilateral hippocampus, compared to sham rats. HI caused a significant reduction in sex-determining region Y-box 2 (Sox2) protein expression at P12 (*p* < 0.05) and P17 (*p* < 0.05), but not at P30 (Fig. 3A). HI also caused a significant reduction in nestin expression at P12 (*p* < 0.01), P17 (*p* < 0.05), and P30 (*p* < 0.05) (Fig. 3B). HI caused a significant decrease in doublecortin (DCX) expression at P12 (*p* < 0.01), P17 (*p* < 0.05), and P30 (*p* < 0.05) (Fig. 3C). HI caused a significant reduction in NeuN expression at P12 (*p* < 0.01), P17 (*p* < 0.05) and P30 (*p* < 0.05) (Fig. 3D). HI caused a significant reduction in calretinin at P17 (*p* < 0.05) and also reduced its expression at P30, but it did not reach statistical significance at that time-point (Fig. 3E); in addition, there was no difference between the groups at P12 (Fig. 3E). HI also caused a significant reduction in calbindin at P30 (*p* < 0.05), but not P12 and P17 (Fig. 3F). In addition, HI caused a significant increase in cleaved PARP (*p* < 0.05) at P12 in the ipsilateral hippocampus, compared to sham rats (Fig. 3G); also, the expression of cleaved poly-ADP-ribose polymerase (PARP) was not different between the groups at P17 and P30. HI reduced GAP-43 at P12, but it did not reach statistical significance at that time-point; HI also caused a significant reduction in GAP-43 at P17 and P30 (*p* < 0.05) (Fig. 3H).

Sildenafil treatment reverted the expression of the early and late neuronal development markers to levels no longer significantly different than sham rats. Sox2 expression was not significantly different than sham rats at P12 and P17 (Fig. 3A). The nestin (Fig. 3B), DCX (Fig. 3C), and NeuN (Fig. 3D) expressions were no longer significantly different than sham rats at P12, P17 and P30. The calretinin expression was not significantly different than sham rats at P17 and P30, and had significantly increased compared to HI rats treated with vehicle at these same time-points (*p* < 0.05) (Fig. 3E). The calbindin expression was not significantly different than sham rats at P30, and had significantly increased compared to HI rats at this same time-point (*p* < 0.05) (Fig. 3F). Sildenafil treatment reverted the levels of cleaved PARP to levels not significantly different than sham rats at P12 (Fig. 3G). GAP-43 expression was not significantly different than sham rats at P17, and had significantly increased compared to HI rats at P12 (*p* < 0.05) (Fig. 3H).

### Sildenafil treatment after HI increased oligodendrogenesis in the hippocampus

**Figure 4:**
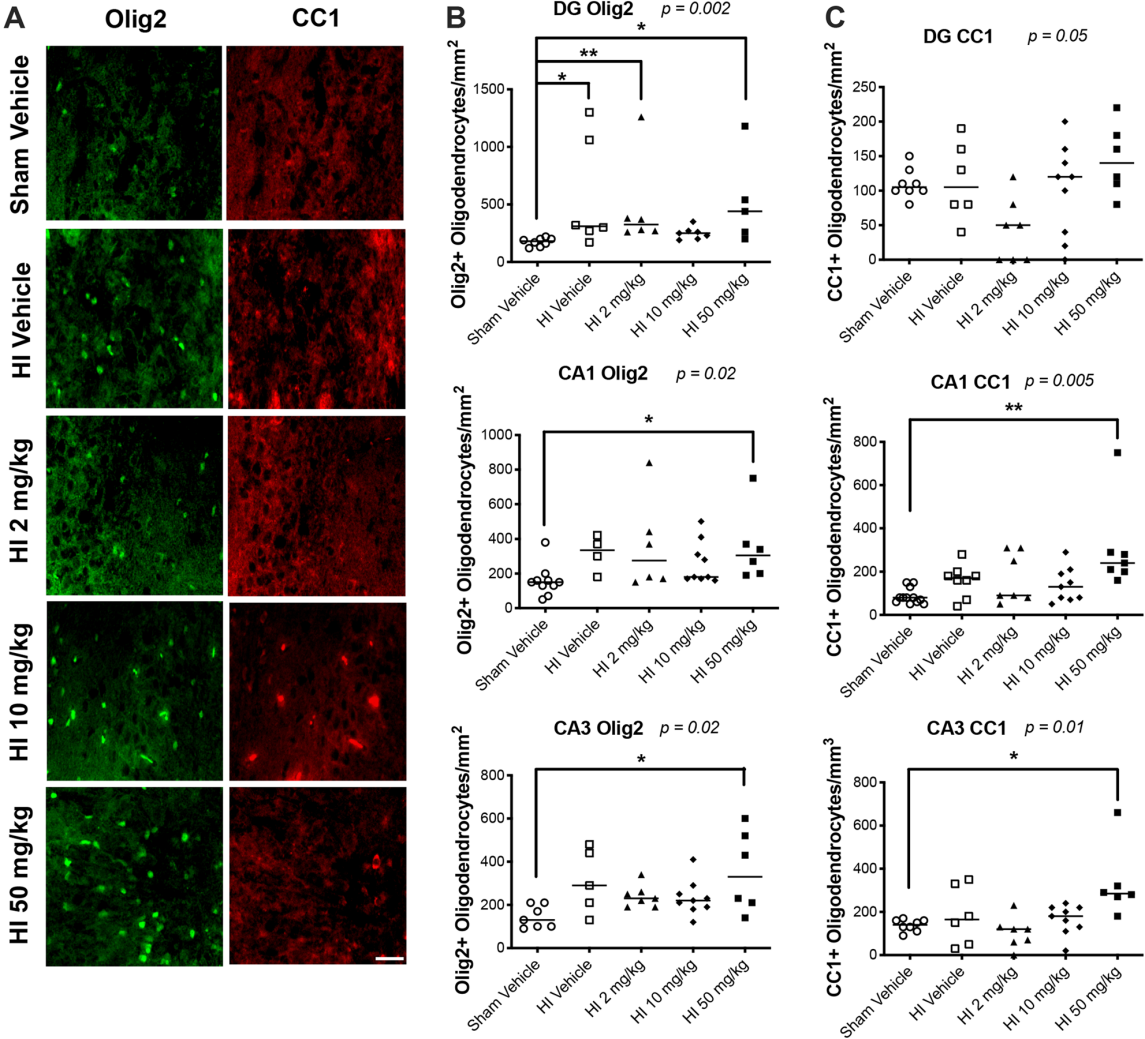
Immunostaining for oligodendrocytes in sham and HI rat pups treated with vehicle or sildenafil. Cells were counted in three regions of the hippocampus: i.e., dentate gyrus, CA1, and CA3 regions of the ipsilateral hippocampus. (**A**) Representative immunofluorescent micrographs of the immature oligodendrocytes labeled with Olig2 and mature oligodendrocytes labeled with CC1 in the ipsilateral CA3 region of the hippocampus. (**B**) Immature oligodendrocytes labeled with Olig2, and (**C**) mature oligodendrocytes labeled with CC1 (median with individual data points representation). Significance: *p* value from Kruskal–Wallis test, with Dunn’s post-hoc comparison tests: *p < 0.05, **p < 0.01. Number of animals: n = 8 sham vehicle, 6 HI vehicle, 7 HI 2 mg/kg, 9 HI 10 mg/kg and 8 HI 50 mg/kg.

When looking more specifically by immunohistochemistry at P30, we found that HI resulted in a significant increase in the number of immature (Olig2+) oligodendrocytes in the DG of the ipsilateral hippocampus (*p* < 0.05), compared to sham rats (Fig. 4A,B). In the CA1 and CA3 regions, we found a trend towards an increase in the number of immature oligodendrocytes in the HI rats treated with vehicle, although this trend did not reach statistical significance compared to sham rats. The number of mature CC1+ oligodendrocytes in the DG, CA1, and CA3 regions of the ipsilateral hippocampus of the HI rats treated with vehicle did not differ from the numbers in sham rats (Fig. 4A,C).

The number of immature Olig2+ oligodendrocytes was significantly higher in the DG region of the HI rats treated with the low-dose (2 mg/kg) of sildenafil and with the high-dose of sildenafil, compared to sham rats. The high-dose of sildenafil also significantly increased the number of immature (Olig2+) oligodendrocytes in both the CA1 and CA3 regions (Fig. 4A,B) (*p* < 0.05), compared to sham rats. In addition, the high-dose of sildenafil increased the number of mature CC1+ oligodendrocytes in both the CA1 (*p* < 0.01) and CA3 (CA3: *p* < 0.05) regions, compared to sham rats (Fig. 4A,C); we observed a similar trend in the DG area, but the difference was not significant between the groups.

### Sildenafil treatment after HI reduced the number of inflammatory cells in the hippocampus

**Figure 5:**
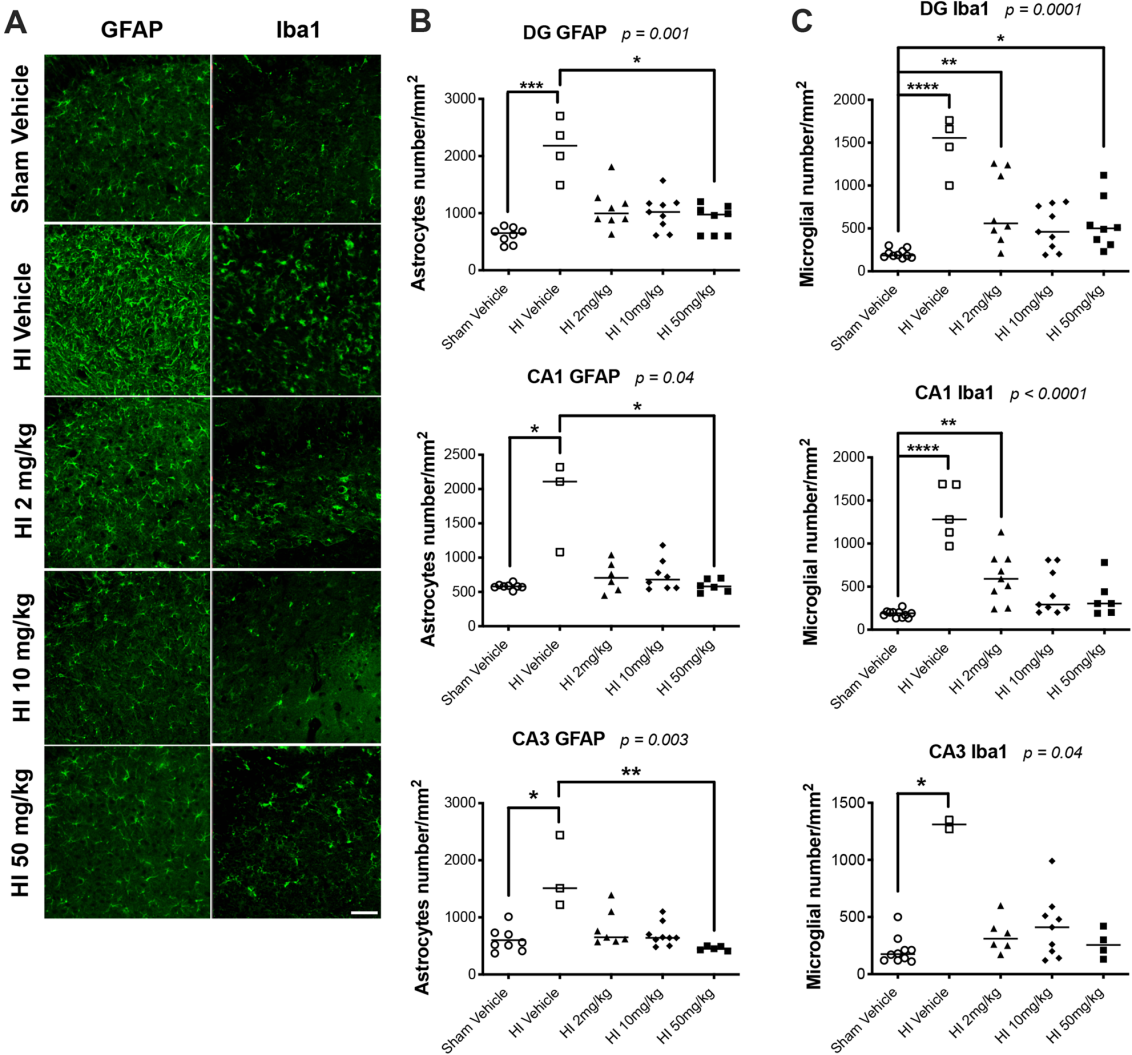
Immunostaining for astrocytes and microglia in sham and HI rat pups treated with vehicle or sildenafil. Cells were counted in three regions of the hippocampus: i.e., dentate gyrus, CA1, and CA3 regions of the ipsilateral hippocampus. (**A**) Representative immunofluorescent micrographs of astrocytes labeled with GFAP and activated microglia labeled with Iba1 in the ipsilateral dentate gyrus. (**B**) Astrocytes labeled with GFAP, and (**C**) activated microglial cells labeled with Iba1 (median with individual data points representation). Significance: *p* value from Kruskal–Wallis test, with Dunn’s post-hoc comparison tests: *p < 0.05, **p < 0.01, ***p < 0.001, ****p < 0.0001. Number of animals: n = 8 sham vehicle, 4 HI vehicle, 8 HI 2 mg/kg, 9 HI 10 mg/kg and 8 HI 50 mg/kg.

HI caused a significant increase in the number of GFAP+ astrocytes in the DG (*p* < 0.001), CA1 (*p* < 0.05), and CA3 (*p* < 0.05) regions of the ipsilateral hippocampus (Fig. 5A,B), compared to sham rats. HI also caused a significant increase in the number of Iba1+ microglia in the DG (*p* < 0.0001), CA1 (*p* < 0.0001), and CA3 (*p* < 0.05) regions (Fig. 5A,C) of the ipsilateral hippocampus, compared to sham rats.

The treatment with sildenafil reduced the astrocytosis in the ipsilateral hippocampus. In the three regions of the hippocampus, all the doses of sildenafil reduced the number of astrocytes to levels not significantly different than sham rats (Fig. 5A,B). In all three regions, the number of astrocytes was significantly lower (*p* < 0.05 for DG and CA1 and *p* < 0.01 for CA3) in the HI rats treated with the high-dose of sildenafil, compared the HI rats treated with vehicle. In addition, the treatment with sildenafil reduced microgliosis. In the DG, the medium-dose of sildenafil reduced the active microgliosis to levels not significantly different than sham rats; the low- and high-doses of sildenafil also reduced the microgliosis, but the number of microglia cells remained significantly higher (respectively, *p* < 0.01; and *p* < 0.05), compared to sham rats (Fig. 5A,C). In the CA1 region, both the medium- and high-doses of sildenafil reduced the active microgliosis to levels not different than sham rats. With respect to the low-dose of sildenafil, the number of microglia cells were reduced but remained significantly higher (*p* < 0.01) than the levels we observed in the sham rats. In the CA3 region, all the doses of sildenafil reduced the active microgliosis to levels not different than sham rats.

### HI decreased the activity of the Pl3kAKT/mTOR pathway in the hippocampus, and sildenafil avoided this effect

**Figure 6:**
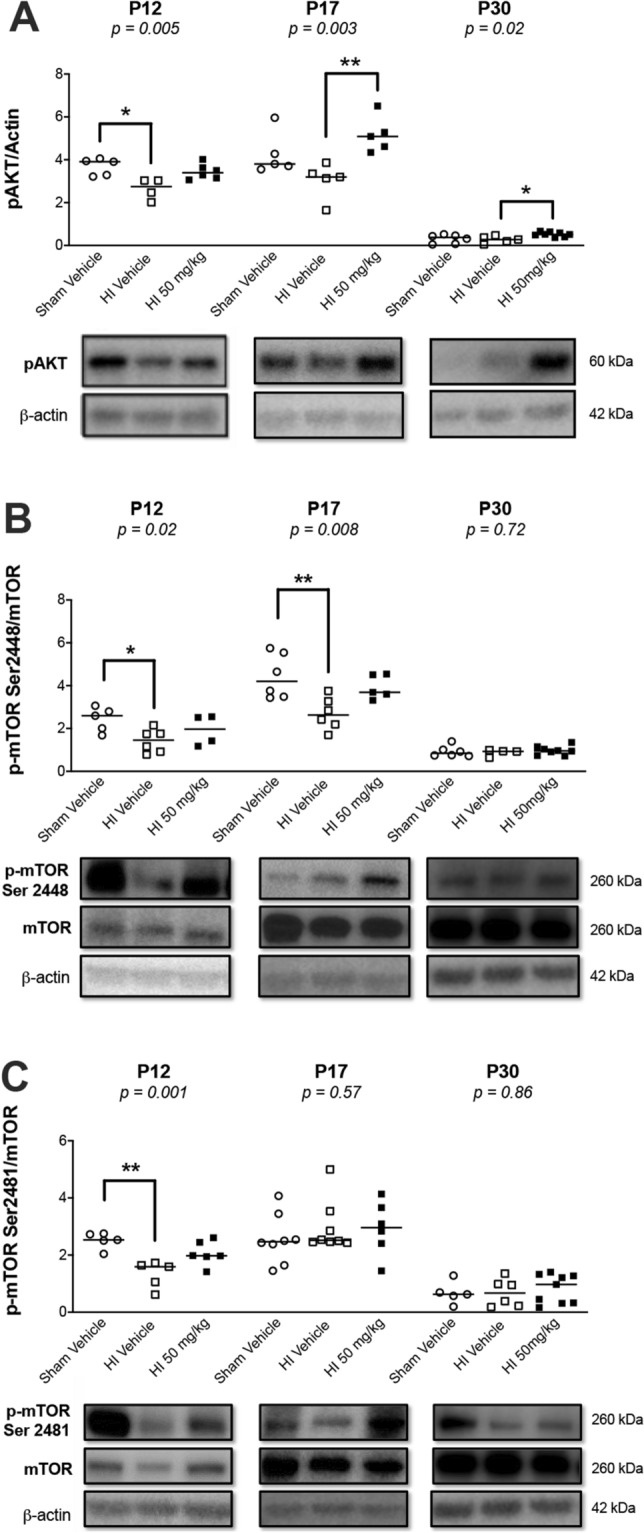
Western blotting for markers of the Pl3k/AKT/MTOR pathway of the ipsilateral hippocampus in sham and HI rat pups treated with vehicle or sildenafil. Median with individual data points representation, with cropped representative western blots. Full-length blots are presented in Supplementary Material. (**A**) p-AKT, (**B**) p-MTOR ser 2448 (mTORC1), and (**C**) p-MTOR ser 2481 (mTORC2). Significance: *p* value from Kruskal–Wallis test, with Dunn’s post-hoc comparison tests *p < 0.05, **p < 0.01. Number of animals: n = 6/8/7 sham vehicle, 6/8/7 HI vehicle, and 7/8/10 HI 50 mg/kg, respectively at P12/P17/P30.

Two days after HI (P12), the p-AKT level decreased significantly (*p* < 0.05), compared to sham rats (Fig. 6A), which was associated with a significant decrease in the levels of mTORC1 (serine 2448) (*p* < 0.05) (Fig. 6B) and mTORC2 (serine 2481) (*p* < 0.01) (Fig. 6C). The levels of mTORC1 (serine 2448) remained significantly decreased (*p* < 0.01) at P17, while the levels of p-AKT and mTORC2 (serine 2481) were not different between the groups. No significant differences occurred at P30.

The sildenafil treatment reverted the levels of p-AKT, mTORC1 (serine 2448), and mTORC2 (serine 2481) at P12 to levels not significantly different than sham rats (Fig. 6A–C). At P17 and P30, the p-AKT level significantly increased in the HI rats treated with sildenafil, compared to HI rats treated with vehicle at these same time-points (*p* < 0.01 at P17 and *p* < 0.05 at P30).

## Discussion

Hippocampal atrophy was a characteristic feature of brain injury 20 days after neonatal HI, associated with a decreased number of mature neurons, an increased number of immature oligodendrocytes, and an increased number of microglia and astrocytes. Similar hippocampal atrophy was previously described in animal models of neonatal HI^13,14^, and has been associated with long-term impairments in learning and memory in human term neonates with neonatal encephalopathy^15,16^. HI also reduced the expression levels of early neuronal markers and late/mature neuronal markers, which suggests the existence of impairments in the normal neuronal development from NSCs to mature neurons, and may explain some of the long-term neurodevelopmental complications encountered by these neonates. In addition, HI increased cleaved PARP, which indicates an increase in caspase-mediated apoptotic cell death following HI, and decreased GAP-43, suggesting that axons also are injured after HI^17^.

**Figure 7:**
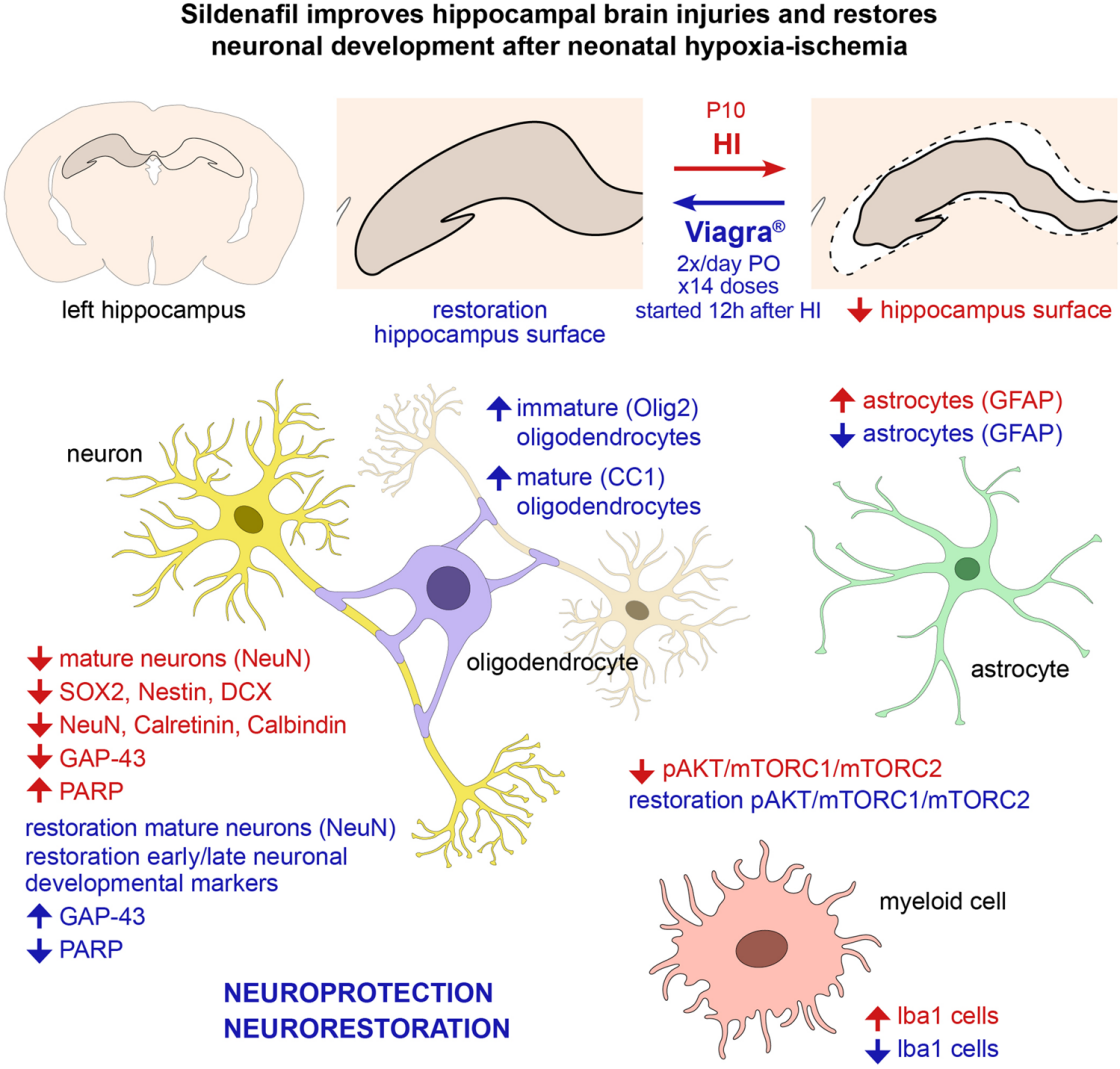
Summary of findings. HI caused significant hippocampal atrophy and a significant reduction of the number of mature neurons, and induced reactive astrocytosis and microgliosis in the hippocampus. HI increased apoptosis and caused significant dysregulation of the normal neuronal development program. Treatment with sildenafil preserved the gross morphology of the hippocampus, reverted the number of mature neurons to levels comparable to sham rats, significantly increased both the immature and mature oligodendrocytes, and significantly reduced the number of microglia and astrocytes. Sildenafil also decreased apoptosis and reestablished the normal progression of post-natal neuronal development. The PI3K/Akt/mTOR pathway, whose activity was decreased after HI in the hippocampus, and restored after sildenafil treatment, may be involved. Sildenafil may have both neuroprotective and neurorestorative properties in the neonatal hippocampus following HI.

Sildenafil treatment started 12 h after HI and continued for 7 days prevented this hippocampal atrophy (Fig. 7). The treatment led to an increase in the number of mature neurons after HI in the neonatal hippocampus to levels not different than sham rats. On the one hand, sildenafil appeared to be *neuroprotective* for the neuronal population by decreasing apoptosis in the secondary phases of injury, and thus neuronal loss in the hippocampus during the acute phase of brain injury. The anti-apoptotic properties of sildenafil have been previously demonstrated in a mouse model of multiple sclerosis^18^. On the other hand, sildenafil appeared to have *neurorestorative* properties after HI by activating the neuronal progenitor cells in the acute to chronic phases of injury, which promoted neurogenesis and neurorestoration in the tertiary phase of injury. The sildenafil treatment increased the levels of early neuronal markers (Sox2, Nestin, and DCX) back to levels not different than sham rats at P12, which suggests that the neural progenitor cell populations and neuroblasts were restored already after only 4 doses of sildenafil. It also is possible that the reduction in apoptosis at P12 may provide neuroprotection for these neural progenitor cell populations^19^. The neural progenitor cells and neuroblasts found mainly between P10–P12 in the dentate gyrus are particularly vulnerable to apoptotic insults^20,21^. Sildenafil has been shown to significantly increase the expression of nestin in the ischemic brain of middle-aged mice^22^. Nestin-expressing adult progenitor cells derived from the subventricular zone are known to be immunoreactive to PDE5^23^. Sildenafil has been shown to improve functional recovery in young rats 28 days post-middle cerebral artery occlusion by significantly increasing the number of immature neurons expressing DCX in the subventricular zone and striatum^4^. Interestingly, even a delayed sildenafil treatment starting 7 days after a focal cerebral ischemia has been found to increase DCX expression in aged rats (3 months), which has led to a functional recovery^24^. Sildenafil has been found to promote the maturation of neuroblasts into granule cells in the hippocampus by enhancing neurotropic support and increasing levels of brain-derived neurotrophic factor (BDNF)^25^.

In our rat model of neonatal hypoxia–ischemia, we found an increase of early neuronal progenitor markers at P12 and P17 after sildenafil treatment following HI, but also an increase of late/mature neuronal markers at P30 in the injured hippocampus. This increase explained the increased number of NeuN-positive cells measured by immunohistochemistry, which probably led to the preservation of the gross morphology of the hippocampus at P30. Sildenafil has been shown to enhance neuronal differentiation by increasing the fraction of mature NeuN-expressing neurons in middle-aged rats treated with subcutaneous sildenafil for 7 days, starting one day after middle cerebral artery occlusion^22^. Sildenafil is known to cross the blood brain barrier and to increase the levels of cyclic guanosine mono-phosphate (cGMP) by inhibiting PDE5 in neurons and glial cells^26^. An elevation of cGMP levels also is thought to directly affect signaling pathways by stimulating the proliferation and differentiation of neural stem cells (NSC) and neural progenitor cells (NPS), which enhances neurogenesis^23^. High levels of cGMP during gestation is correlated with preferential differentiation of neural stem cells into neurons, compared to glial cells^27^. In our study, sildenafil treatment appeared to revert the effect of HI by promoting normal brain development, but it did not increase neurogenesis beyond sham levels. After sildenafil treatment, a similar increase in mature neurons was found in the cortex of the same animal model near the infarct boundary zone^8^, and it is possible that the new neurons may have migrated to the cortex from the hippocampus after ischemic brain injury^28^. Interestingly, the activation of neurogenesis was associated with an increase in GAP-43, a protein known to increase during axon remodeling and during synaptic regeneration after injury^17,28^. Further work is required to understand the potential role of sildenafil on synaptic formation and plasticity of the neonatal brain after HI.

In our study, sildenafil treatment also increased the number of immature and mature oligodendrocytes. Similar to neurons, oligodendrocytes have been shown to play a crucial role in neurorestoration after HI^29^. Although oligodendrocytes are highly susceptible to HI insult in the central nervous system, HI also initially provides them with proliferation signals to initiate repair^30^. Interestingly, in our study, we observed an increase of immature oligodendrocytes in the dentate gyrus of the HI rats treated with vehicle, which suggests that the endogenous repair processes was activated. However, this increase was not observed in the other areas of the hippocampus and was not associated with an increase of mature oligodendrocytes, which probably indicates that these cells, even if they may have been activated initially, would not produce functional oligodendrogenesis without additional help. Sildenafil treatment may potentiate intrinsic repair mechanisms and may lead to the repair of white matter injury following HI. Sildenafil has been shown to increase myelin sheath thickness in the peripheral nervous system, thereby improving peripheral neuropathy in adult diabetic mice^31^. Sildenafil also has been found to increase oligodendroglial progeny in the ischemic brain of middle-aged mice by amplifying nestin-expressing neural stem cells^22^. Further studies should investigate in more detail the white matter of these animals after HI and sildenafil treatment.

The neurogenesis and the oligodendrogenesis we observed after sildenafil treatment were associated with a reduction in inflammatory cells 20 days after HI. While acute neuroinflammation may be part of an endogenous trial of repair that may be activated in the hippocampus after neonatal HI, chronic and persistent neuroinflammation typically is associated with poorer performance on neurodevelopmental testing^14^. Chronic and persistent neuroinflammation are known to be a potent contributor to white matter injury through axonal and myelin degeneration^32^. In our study, hippocampal gliosis was a persistent feature still evident 20 days after HI. Reactive astrocytosis and activated microgliosis were evident across the hippocampus after HI, and sildenafil treatment significantly reduced astrocytosis and activated microgliosis across the hippocampus. Reducing microgliosis has been shown to be beneficial in reducing the severity of cortical and hippocampal injuries in rodent models of neonatal HI^33^. Reducing reactive astrocytosis may reduce inflammatory cytokine release^33^. This reduction in neuroinflammation in the tertiary phase of injury may create a healthier neuronal niche for neuroregeneration, and thus needs to be further investigated in the model of neonatal HI after sildenafil administration.

The neuroprotective, neurorestorative, and anti-inflammatory mechanism of action of sildenafil remains to be elucidated in the rat model of neonatal HI. A possible molecular pathway that could be involved in these beneficial effects is the PI3K/Akt/mTOR pathway, whose activation is known to increase neurogenesis, regulate myelination, and influence neuroinflammation^11,34–36^. mTOR is composed of two functionally distinct complexes^37^. Active mTOR complex 1 (mTORC1) promotes the maturation and survival of NSCs, increases cellular proliferation, and suppresses apoptosis^38^. Active mTORC2 promotes cell survival, and enhances neuronal and oligodendrocyte differentiation^39–41^. In our study, HI significantly decreased the levels of phosphorylated AKT, mTORC1, and mTORC2 in the hippocampus, whereas sildenafil treatment reverted their levels to levels not significantly different than sham rats. The intracellular increase in cGMP levels induced by sildenafil activates PI3K, which activates AKT by phosphorylation. Sildenafil has been shown to increase SVZ cell proliferation by increasing AKT and glycogen synthase kinase 3 (GSK-3) phosphorylation in male Wistar adult rats^42^. The increase in p-AKT confers neuroprotection by protecting neurons against apoptosis in cortical, hippocampal, and motor neuronal cultures^43^. The AKT pathway also enhances Sox2 stability and activity in mouse embryonic stem cells (ESCs), which is critical for self-renewal and pluripotency qualities of NSC^44^. The increase in p-AKT expression leads to increased mTORC1 levels, which has been shown to be critical for NSCs differentiation in the adult and aging forebrain^45^, and for neuronal survival following transient focal cerebral ischemia in mice^46^. Thus, a dysregulation of the Pl3K/AKT/mTOR pathway possibly explains the abnormalities in neurogenesis and oligodendrogenesis we observed after HI at term-equivalent age.

In this study, we decided to wait 12 h after HI before starting sildenafil treatment (rather than giving immediately after HI) to ensure that the secondary phase of injury after HI had already started, and to test the potential neurorestorative effect of sildenafil in addition to its neuroprotective effect. The fact that sildenafil had such a beneficial effect even when started 12 h after HI insult contrasts with other previously tested neuroprotective strategies (such as melatonin and Xenon) that were found effective only when given before, during, or just after the HI insult in rat model of neonatal HI^47–49^. For example, hypothermia treatment started 12 h after HI in a P7 Vannucci model offered no benefit to rats with moderate HI injury and was even deleterious to rats with severe HI injury^50^. Only erythropoietin demonstrated similar beneficial effects when started with a delay after HI^51^. In our study, even if sildenafil treatment was stopped after 7 days, its effects persisted beyond the 7-day treatment and remained evident at P30. We administered the sildenafil treatment by oral route instead of the intraperitoneal or subcutaneous route described in most previous animal experiments^22^, because oral sildenafil is the most commonly used route with human neonates. Based on our histology and immunohistochemistry results in the hippocampus, the dose of 10 mg/kg of sildenafil appeared as beneficial as the high-dose (50 mg/kg) of sildenafil. However, only the dose of 50 mg/kg of sildenafil was used for western blots, since sildenafil provided a clear dose-dependent beneficial effect on the cerebral cortex and retina, with the 50 mg/kg being the most efficient dose^8,9^. Apoptosis was only tested at P12 and not sooner to ensure the treated rats pups had received 3 doses of study drug before sacrifice and thus to ensure to test the effect of sildenafil, even if 48 h after HI may be on the late side when examining apoptosis associated with initial HI injury.

The experiments described here were only performed in male rat pups considering the previously described limited brain repair in males compared to females^52^, with the idea to test the beneficial effect of the study drug first in the population less inclined to repair processes. It will be important in the future to further test the potential sex difference in response to sildenafil treatment, since pathways activation after HI and sildenafil responses may be sex-dependent^53^. In addition, it will be essential to test sildenafil treatment in combination with therapeutic hypothermia to allow for further translation in high-resource settings; these treatments may have synergistic effects, for example, on apoptosis^54^. Future functional experiments such as the novel object recognition test should also be planned to determine whether our findings translate to improvements in memory; it was previously demonstrated that the structural improvements conferred by sildenafil translate to functional improvements in gait and vision^8,9^.

In conclusion, the hippocampus is a vulnerable target in the rat model of neonatal HI. Sildenafil is a promising novel treatment for such brain injury following HI at term-equivalent age. Sildenafil may have both neuroprotective and neurorestorative roles. Sildenafil decreased apoptosis and re-established the normal progression of post-natal neuronal development program in the hippocampus; sildenafil also enhanced the maturation of oligodendrocytes, and reduced the chronic neuroinflammation in the hippocampus. All these effects lead to the preservation of the hippocampal structure after HI. The PI3K/Akt/mTOR pathway may be involved in these neuroprotective and neurorestorative roles of sildenafil.

## Methods

### Animals and experimental design

Our experiments were in accordance with the standard operating procedures and guidelines for the use of animals in research as per the Canadian Council on Animal Care Guide for the Care and Use of Experimental Animals, and the Animals for Research Act. They were approved by the local Animal Care Committee from the Montreal Children’s Hospital, McGill University Health Centre. The study is reported according to ARRIVE guidelines. Adult female Long–Evans rats with their male-only litters (Charles Rivers Laboratories) were received in the animal facility, housed under standard environment, and allowed food and water ad libitum.

### Induction of neonatal HI

To mimic the brain injuries observed in human asphyxiated neonates at term-equivalent age, we used the well-established Vannucci rat model of neonatal HI at term-equivalent age^55–57^. This model combines ischemia (left common carotid artery ligation) and a 2-h exposure to hypoxia (8% oxygen) in 10-day-old (P10) male Long–Evans rat pups. The rats undergoing the whole procedure were considered as the HI group. Sham-operated rat pups (identical procedure as the HI group, but without ligation and hypoxia) served as the control group. The sample size of the current study was a size of convenience subject to the restrictions imposed by the institutional ethics committee that reviewed it, and based on the principles of the 3 Rs (replacement, reduction, and refinement). Normal core body temperature (36 °C) was maintained in both the sham and HI rat pups, while they were separated from the mother during the whole procedure (surgery ± hypoxia), by using warming blankets (Cincinnati Sub-Zero, Cincinnati, USA).

### Sildenafil preparation and administration

Sildenafil (Viagra^®^, 100-mg tablets; Pfizer) was prepared in a way similar to when it is given to human neonates for persistent pulmonary hypertension^8,9^. HI and sham-operated rat pups were weighed every day and randomized to sildenafil or vehicle twice daily by oral gavage, starting 12 h after HI and continuing for 7 consecutive days from P11 to P17. For histology and immunohistochemistry (n = 8–10 rat pups in each group), three different doses of sildenafil were used with the HI rat pups—a low-dose (2 mg/kg), a medium-dose (10 mg/kg), and a high-dose (50 mg/kg)—which corresponded somewhat to the equivalent recommended amount for human neonates, and accounted for the differences between human and rat metabolism^58^. For western blot (n = 6–10 rat pups in each group), only the high-dose (50 mg/kg) of sildenafil was used, since it had been found to have the most beneficial effects on the cerebral cortex and retina^8,9^.

### Hippocampal size

At P30, the rats were euthanized with an intraperitoneal injection of sodium pentobarbital (100 mg/kg) and then transcardially perfused. The brains were extracted, post-fixed, and serially sectioned. Sections were collected in the hippocampus area. After hematoxylin and eosin staining using a standard protocol, the sections were examined with a light microscope with a 5 × objective. For each section, overlapping microphotographs were captured using a digital camera attached to the microscope. To obtain pictures of the entire coronal section, the pictures were stitched using a panoramic image stitching software. The surfaces of the ipsilateral and contralateral hippocampus were blindly measured on two sections by one investigator, who was blind to the treatment group subdivision. Measurements were averaged to represent each animal. To remove any potential effects of individual body weight, the ratios between ipsilateral and contralateral hippocampi were calculated and used for comparison between the groups.

### Neurons, oligodendrocytes, astrocytes, and microglia’s count

Immunohistochemistry was performed at P30. Staining with a primary antibody was performed to inspect for mature neurons by labeling with an anti-neuronal nuclei (mouse anti-NeuN, MAB377; Millipore, Burlington, Massachusetts, USA; dilution 1:500, incubation time 1 h at room temperature), for immature oligodendrocytes by labeling with an oligodendrocyte transcription factor (Olig2) (rabbit anti-Olig2, ab109186; Abcam, Cambridge, UK; dilution 1:500, incubation overnight at 4 °C), for mature oligodendrocytes by labeling with an anti-adenomatous polyposis coli clone (CC1) (mouse anti-CC1, ab16794; Abcam, Cambridge, UK; dilution 1:1000, incubation overnight at 4 °C), for astrocytes by labeling with a glial fibrillary acidic protein (GFAP) (mouse anti-GFAP, SMI-22R; Covance, Princeton, New Jersey, USA; dilution 1:1000, incubation overnight at 4 °C), and for microglia by labeling with an ionized calcium binding adaptor molecule 1 (Iba-1) (rabbit anti-Iba1, 019-19741; Wako Pure Chemical Industries, Osaka, Japan; dilution 1:600, incubation overnight at 4 °C). Then, sections were incubated with their respective secondary antibodies. The stained brain sections were visualized using a light microscope with a 20 × objective, and pictures were taken in three regions of the hippocampus: i.e., dentate gyrus, cornu ammonis (CA) 1, and the CA3 regions of the ipsilateral hippocampus. Number of neurons (NeuN), oligodendrocytes (CC1, Olig2), astrocytes (GFAP), and microglia (Iba1) per mm2 were calculated in these three regions of the ipsilateral hippocampus for each animal. One investigator took pictures of the respective fields of view, and another investigator, who was blind to the treatment group subdivision, assessed the cell counts.

### Markers of early/late neuronal development, apoptosis, axonal regeneration, and mTOR pathway

Markers of early/late neuronal development, apoptosis, axonal regeneration, and the mTOR pathway were studied at three different time-points—P12, P17, and P30—using western blot. Brains were dissected, and the ipsilateral hippocampal tissue was extracted and flash frozen in liquid nitrogen and stored at − 80 °C. The samples were lysed by sonification. Lysates were centrifuged, and their protein concentration was determined by a BCA protein assay kit (23225). Protein samples were diluted with a Laemmli buffer (S3401) and distilled water to obtain 15 μg of protein per sample. Proteins were separated through gel electrophoresis, and then the gels were transferred to a Polyvinylidene difluoride membrane (10600023). Protein blots were incubated with the primary antibodies. More specifically, we tracked the protein expression profile of the neural development markers as they differentiated and matured. For early neuronal markers, we measured the expression of the sex-determining region Y-box 2 (Sox2) (rabbit anti-Sox2, ab97959; Abcam, Cambridge, UK; dilution 1:1000), nestin (mouse anti-nestin, MAB353; Millipore, Burlington, Massachusetts, USA; dilution 1:1000), and doublecortin (DCX) (rabbit anti-DCX, ab18723; Abcam, Cambridge, UK; dilution 1:1000). For late neuronal markers, we used calretinin (mouse anti-calretinin, 6B3pur; Swant, Marly, Fribourg, Switzerland; dilution 1:1000) as a marker for younger post-mitotic neuronal populations, calbindin (rabbit anti-Calbindin, CB 38; Swant, Marly, Fribourg, Switzerland; dilution 1:500) as a marker for mature granular cells^59^, and NeuN (mouse anti-NeuN, MAB377; Millipore, Burlington, Massachusetts, USA; dilution 1:500) as a marker for mature neurons^60^. To study apoptosis, we quantified levels of the 89 kDa cleaved poly-ADP-ribose polymerase (PARP) (rabbit anti-PARP, 9542; Cell Signaling Technology, Danvers, Massachusetts, USA; dilution 1:1000) that is associated with apoptotic cell death. To determine whether the neuronal changes translated to the axonal level, we also measured the levels of the anti-growth associated protein 43 (GAP-43) (Sheep anti-GAP-43, NBP1-41123; Novus Biologicals, Centennial, Colorado, USA; dilution 1:1000), a protein expressed at high levels in neuronal growth cones during neuronal development and also highly expressed during axonal regeneration. To evaluate the activity of the Pl3k/AKT/mTOR pathway, we quantified the levels of phosphorylated protein kinase B (AKT) (rabbit anti-phospho-Akt Ser473 D9E, 4060; Cell Signaling Technology, Danvers, Massachusetts, USA; dilution 1:1000); the levels of phospho-mTOR serine 2448 (rabbit anti-p-mTOR Ser2448, 2971; Cell Signaling Technology, Danvers, Massachusetts, USA; dilution 1:1000), which reflect the mammalian target of rapamycin complex 1 (mTORC1) activity; the levels of phospho-mTOR Ser2481 (rabbit anti-p-mTOR Ser2481, 2974; Cell Signaling Technology, Danvers, Massachusetts, USA; dilution 1:1000), which reflect the mammalian target of rapamycin complex 2 (mTORC2) activity; and the levels of pan mTOR antibody (rabbit anti-mTOR, 2972; Cell Signaling Technology, Danvers, Massachusetts, USA; dilution 1:1000). Following the primary incubation, we incubated the membranes their respective secondary antibodies. We analyzed the protein blots using Image Lab^®^ software, and we quantified their band absorbance. We used actin and total protein levels (for phosphorylated proteins) expression as housekeeping proteins for normalization.

### Data analysis

For the analysis, we subdivided the HI rat pups into the randomly attributed treatment groups: vehicle (0 mg/kg) or sildenafil (2, 10 and 50 mg/kg for histology and immunohistochemistry and 50 mg/kg for western blots). We assessed statistical differences in outcomes between the different treatment groups with Kruskal–Wallis non-parametric tests; pairwise comparisons were conducted to compare the different groups of HI rats to sham rats treated with vehicle, as well as compare the sildenafil-treated rats to HI rats treated with vehicle. For multiple comparisons, we applied the Dunn’s post hoc comparison tests to adjust the α-level as necessary. We considered *p* value < 0.05 as statistically significant. We performed all the statistical analyses with GraphPad Prism^®^ (GraphPad Software Inc, San Diego, CA, USA).

## Supplementary Information

Supplementary Figures.

## Abbreviations

CA: Cornu ammonis
cGMP: Cyclic guanosine monophosphate
DG: Dentate gyrus
HI: Hypoxia–ischemia
mTOR: Mammalian target of rapamycin
NPS: Neural progenitor cells
NSCs: Neural stem cells
P: Postnatal
PDE5: Phosphodiesterase type 5
SGZ: Subgranular zone
