# Supplementary material for: Sildenafil improves hippocampal brain injuries and restores neuronal development after neonatal hypoxia–ischemia in male rat pups
Source: Sci Rep. 2021 Nov 11;11:22046. doi: 10.1038/s41598-021-01097-6 (PMC8586032; doi:10.1038/s41598-021-01097-6)
Supplement: Supplementary file 1 — Supplementary Figures. [file 41598_2021_1097_MOESM1_ESM.pdf]

# Sox2

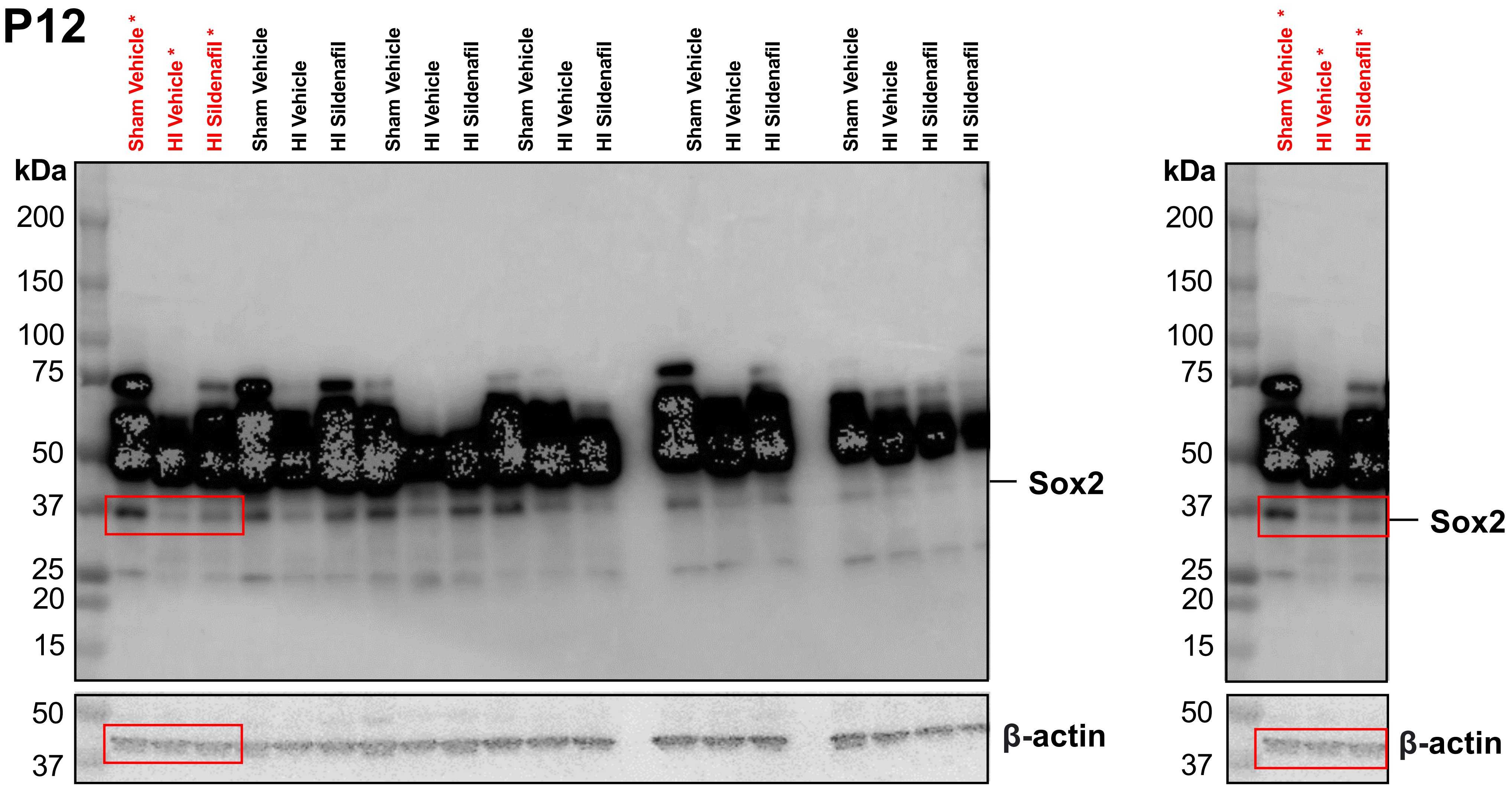


**FIGURE 3A – P12: Western blotting for Sox2 at P12**, showing full-length western blots (on left panel) and representative samples chosen to be cropped for main Figure 3 (on right panel). The regions of the original blots used in main figure have been denoted using red boxes.

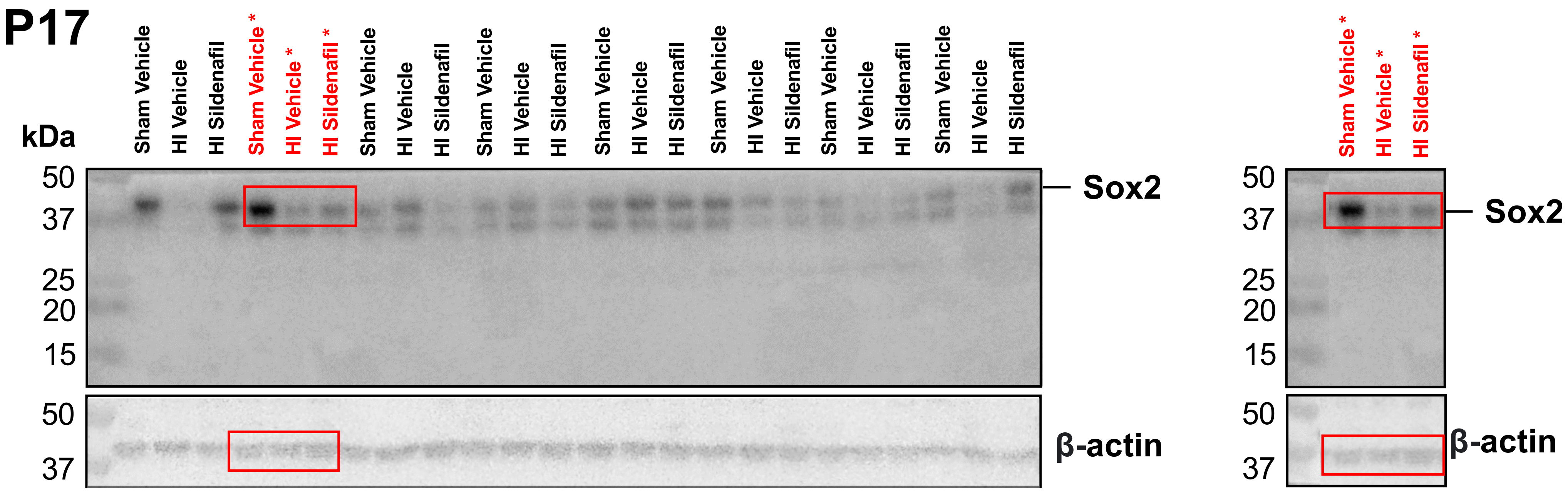


**FIGURE 3A – P17: Western blotting for Sox2 at P17**, showing full-length western blots (on left panel) and representative samples chosen to be cropped for main Figure 3 (on right panel). The regions of the original blots used in main figure have been denoted using red boxes.

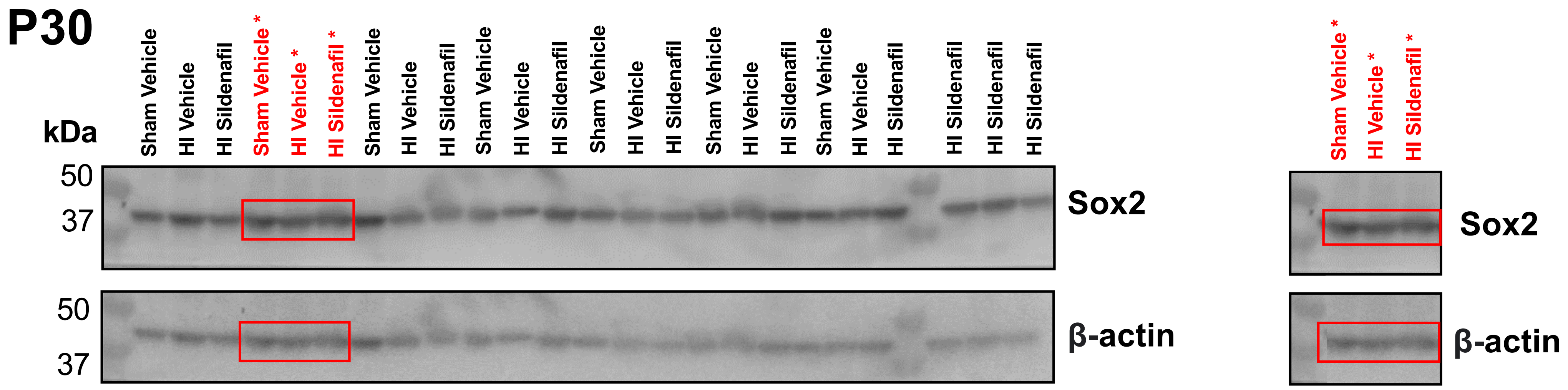


**FIGURE 3A – P30: Western blotting for Sox2 at P30**, showing full-length western blots (on left panel) and representative samples chosen to be cropped for main Figure 3 (on right panel). The regions of the original blots used in main figure have been denoted using red boxes.

# Nestin

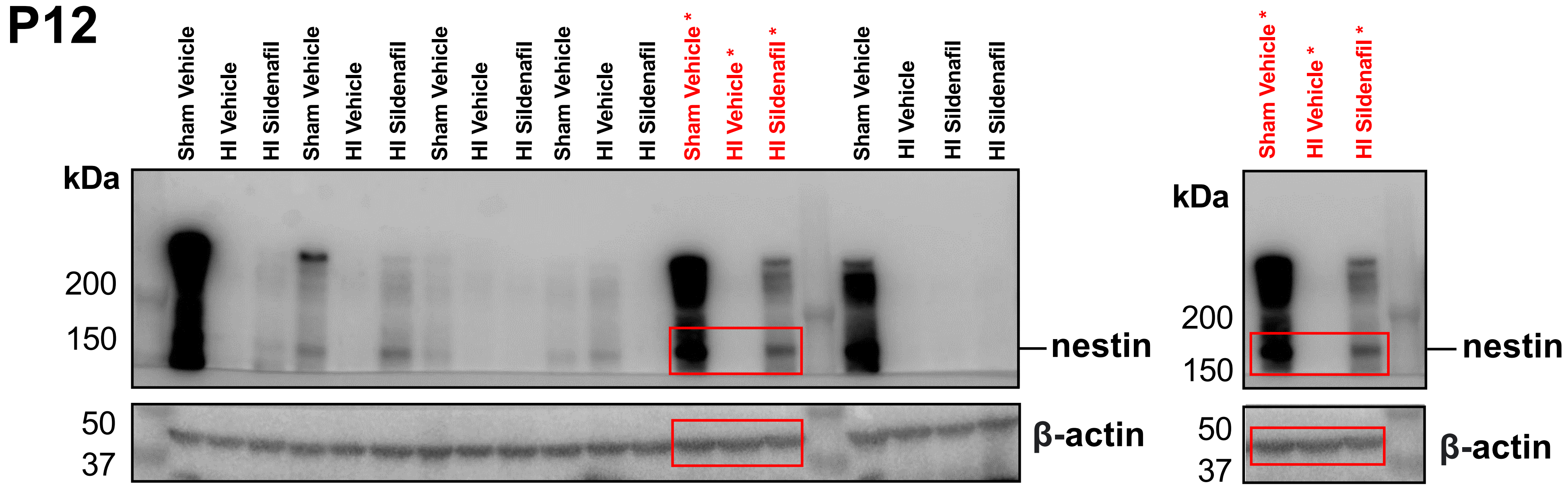


**FIGURE 3B – P12: Western blotting for Nestin at P12**, showing full-length western blots (on left panel) and representative samples chosen to be cropped for main Figure 3 (on right panel). The regions of the original blots used in main figure have been denoted using red boxes.

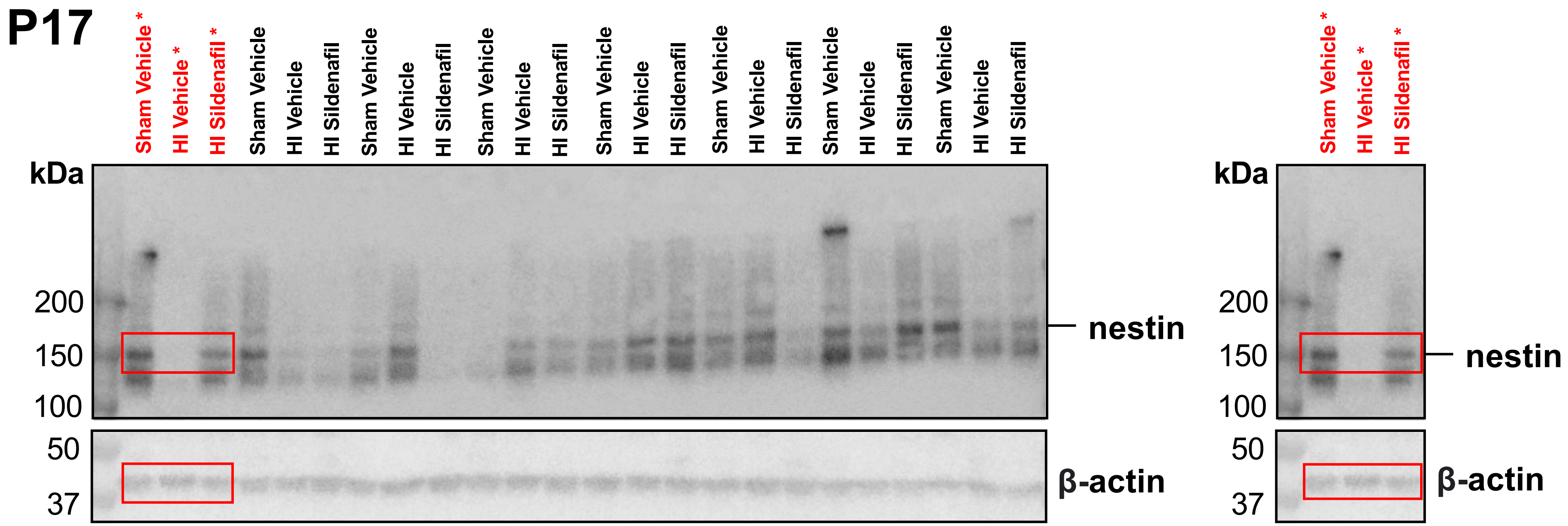


**FIGURE 3B – P17: Western blotting for Nestin at P17**, showing full-length western blots (on left panel) and representative samples chosen to be cropped for main Figure 3 (on right panel). The regions of the original blots used in main figure have been denoted using red boxes.

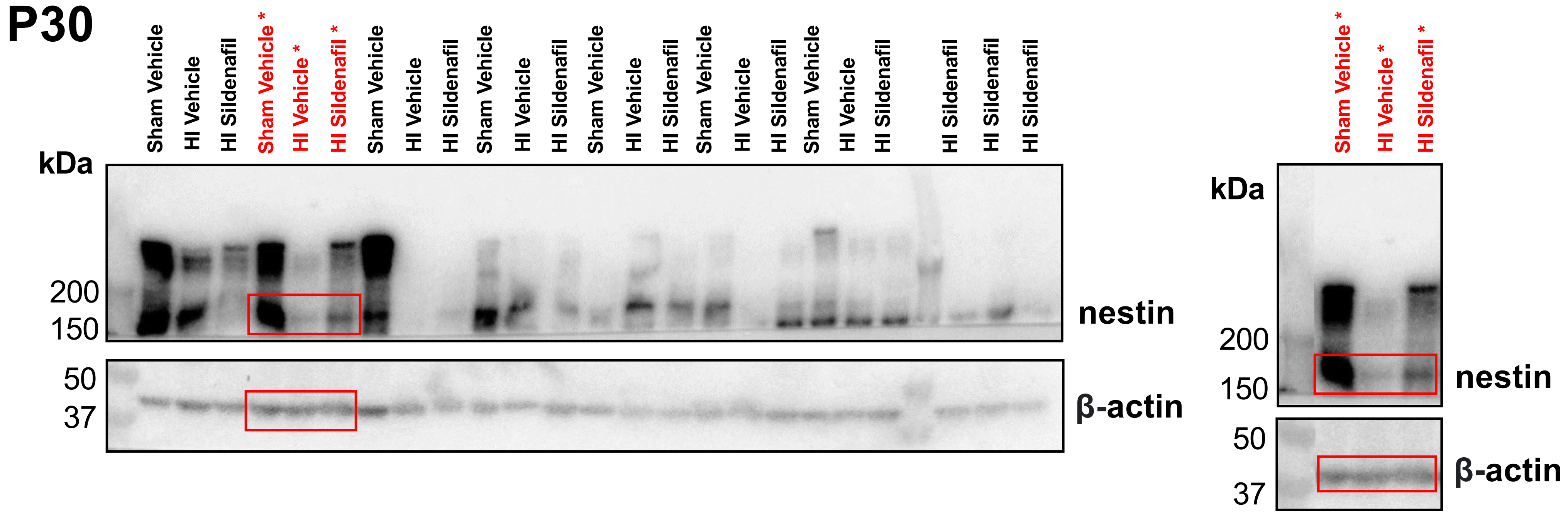


**FIGURE 3B – P30: Western blotting for Nestin at P30**, showing full-length western blots (on left panel) and representative samples chosen to be cropped for main Figure 3 (on right panel). The regions of the original blots used in main figure have been denoted using red boxes.

# DCX

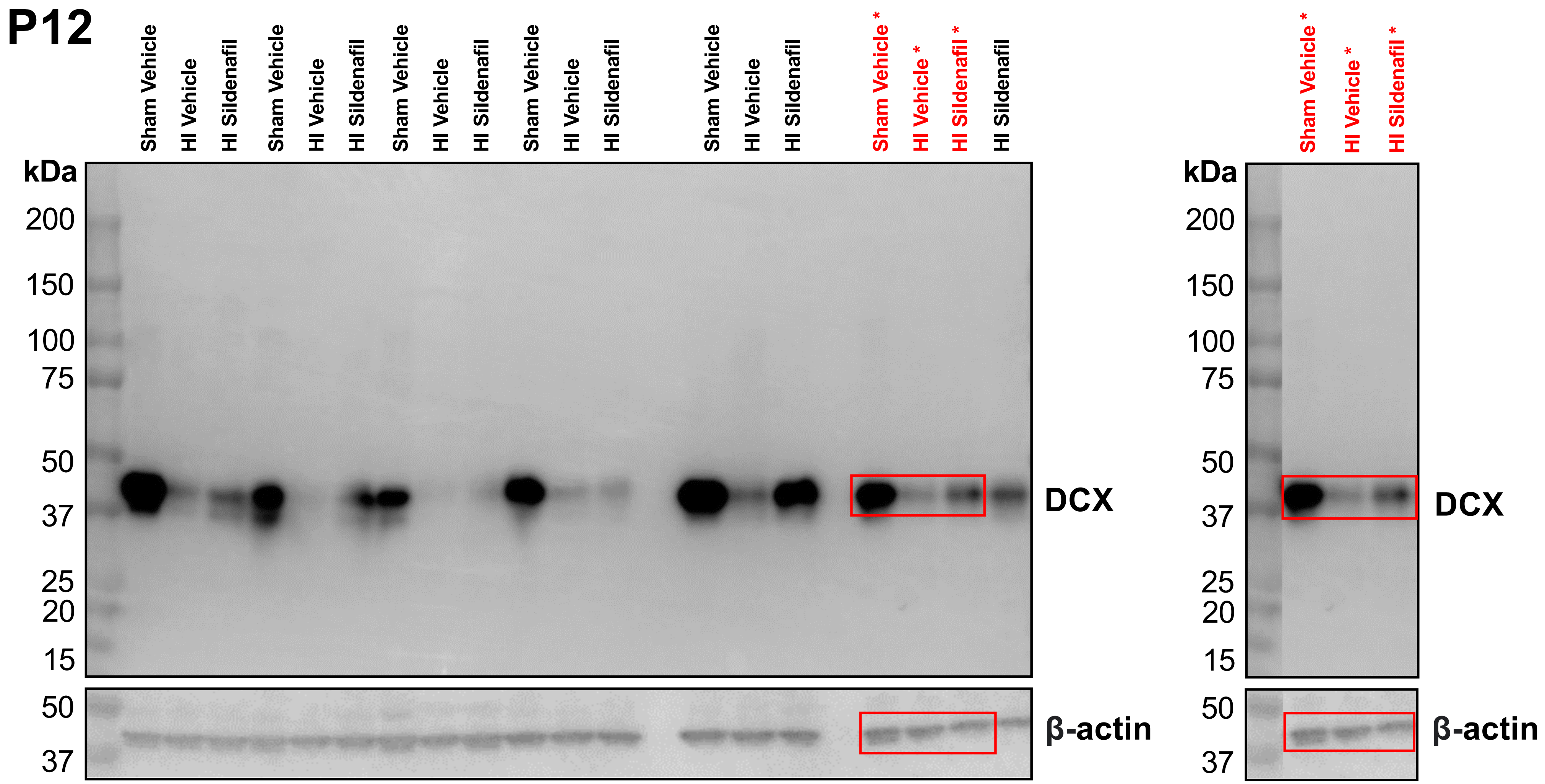


**FIGURE 3C – P12: Western blotting for DCX at P12**, showing full-length western blots (on left panel) and representative samples chosen to be cropped for main Figure 3 (on right panel). The regions of the original blots used in main figure have been denoted using red boxes.

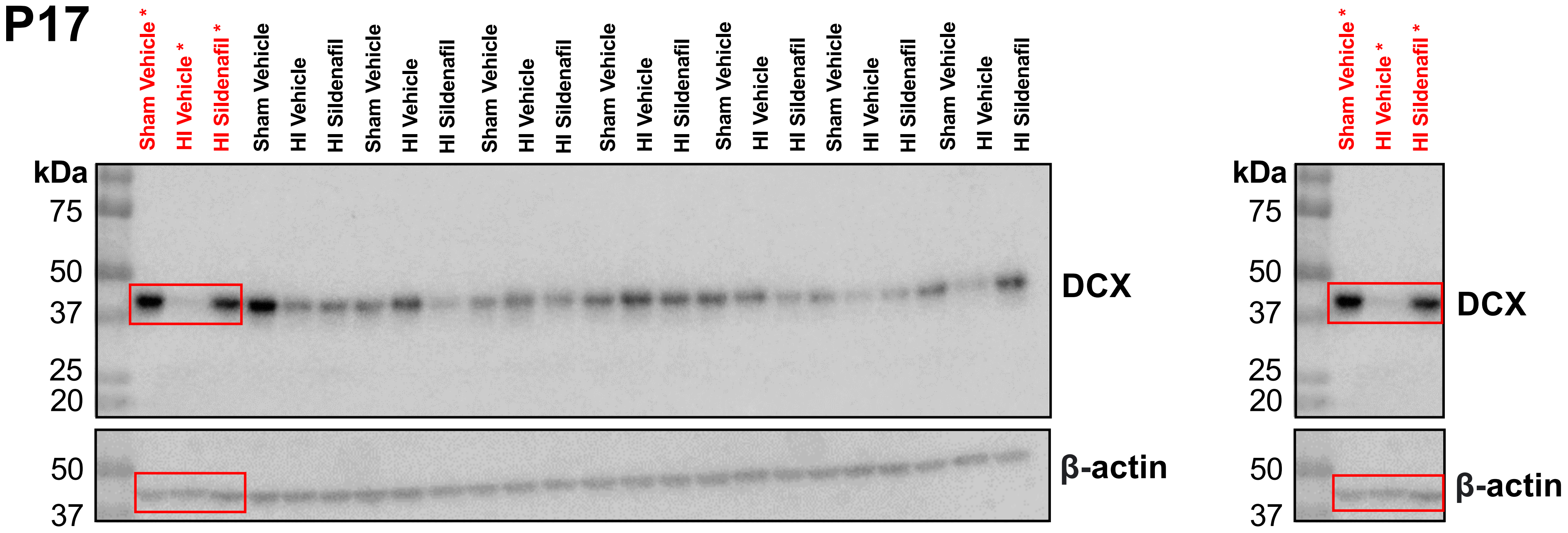


**FIGURE 3C – P17: Western blotting for DCX at P17**, showing full-length western blots (on left panel) and representative samples chosen to be cropped for main Figure 3 (on right panel). The regions of the original blots used in main figure have been denoted using red boxes.

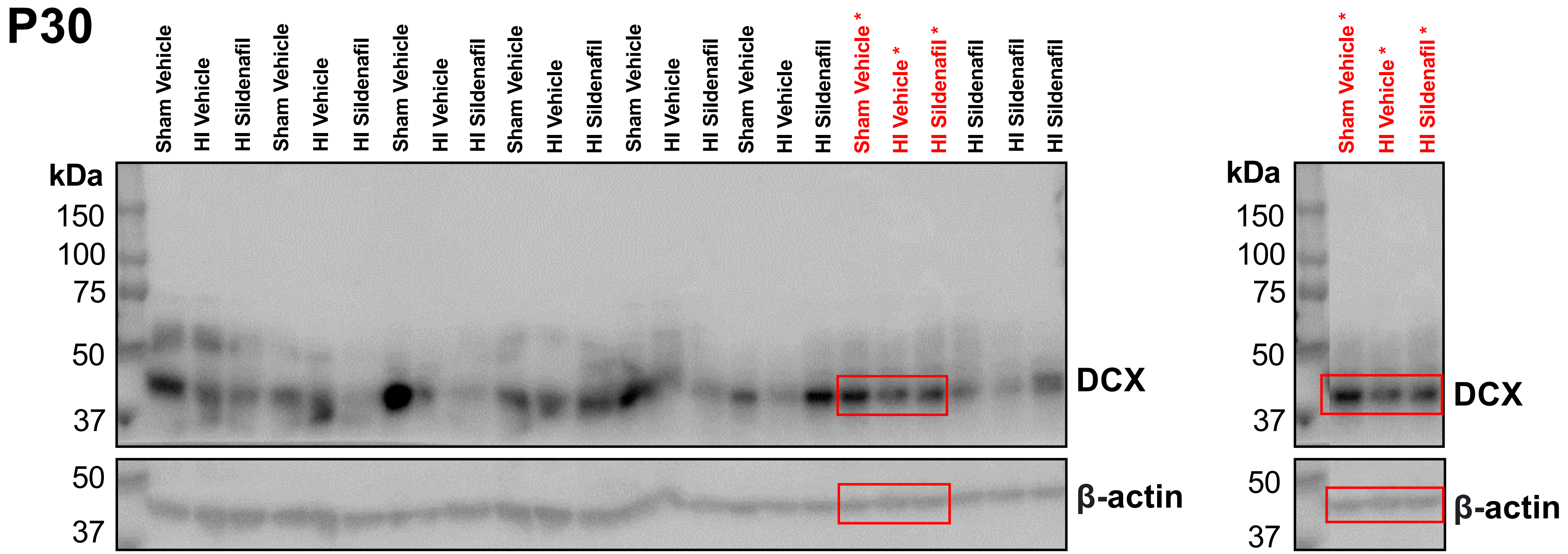


**FIGURE 3C – P30: Western blotting for DCX at P30**, showing full-length western blots (on left panel) and representative samples chosen to be cropped for main Figure 3 (on right panel). The regions of the original blots used in main figure have been denoted using red boxes.

# NeuN

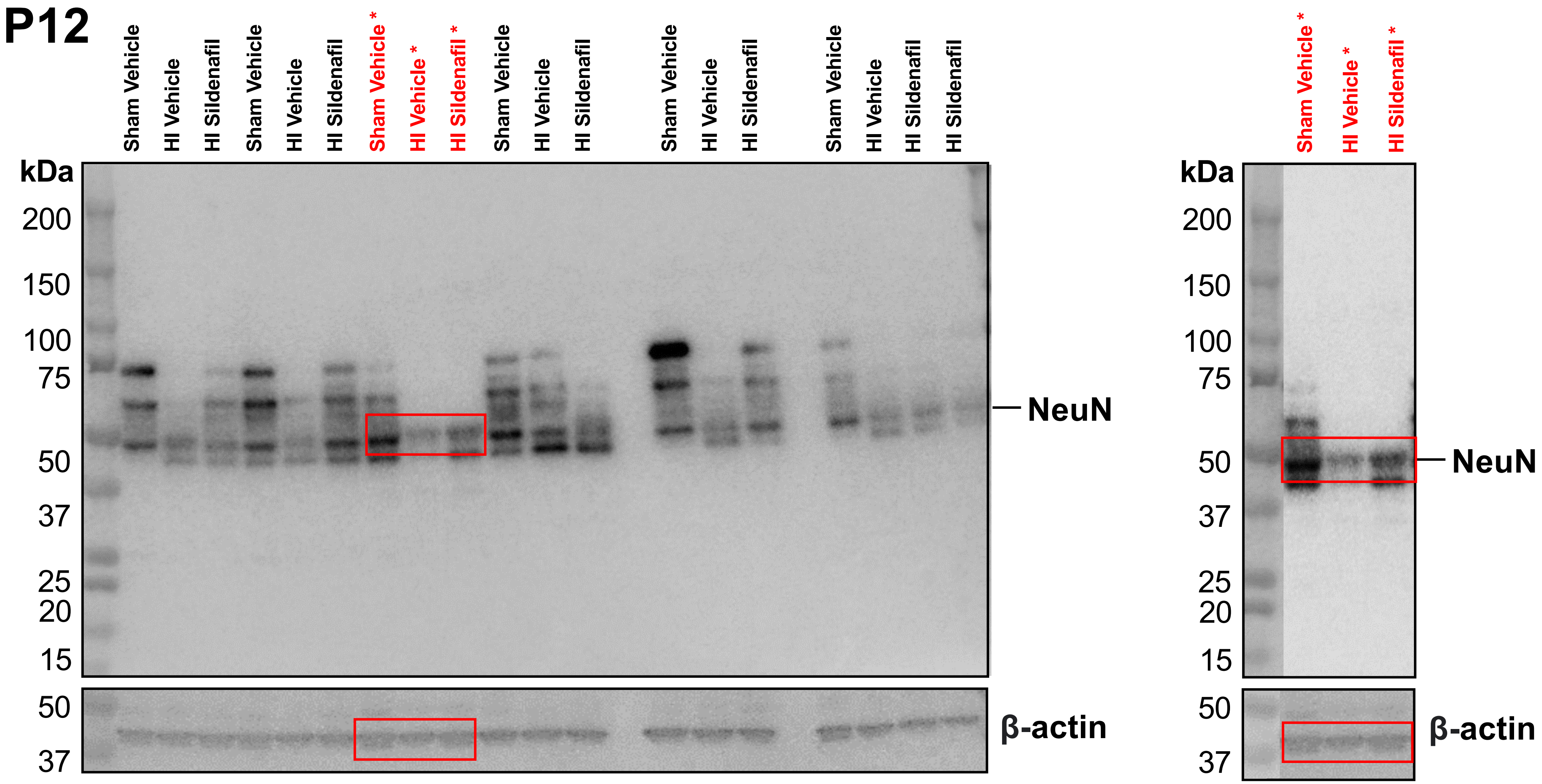


**FIGURE 3D – P12: Western blotting for NeuN at P12**, showing full-length western blots (on left panel) and representative samples chosen to be cropped for main Figure 3 (on right panel). The regions of the original blots used in main figure have been denoted using red boxes.

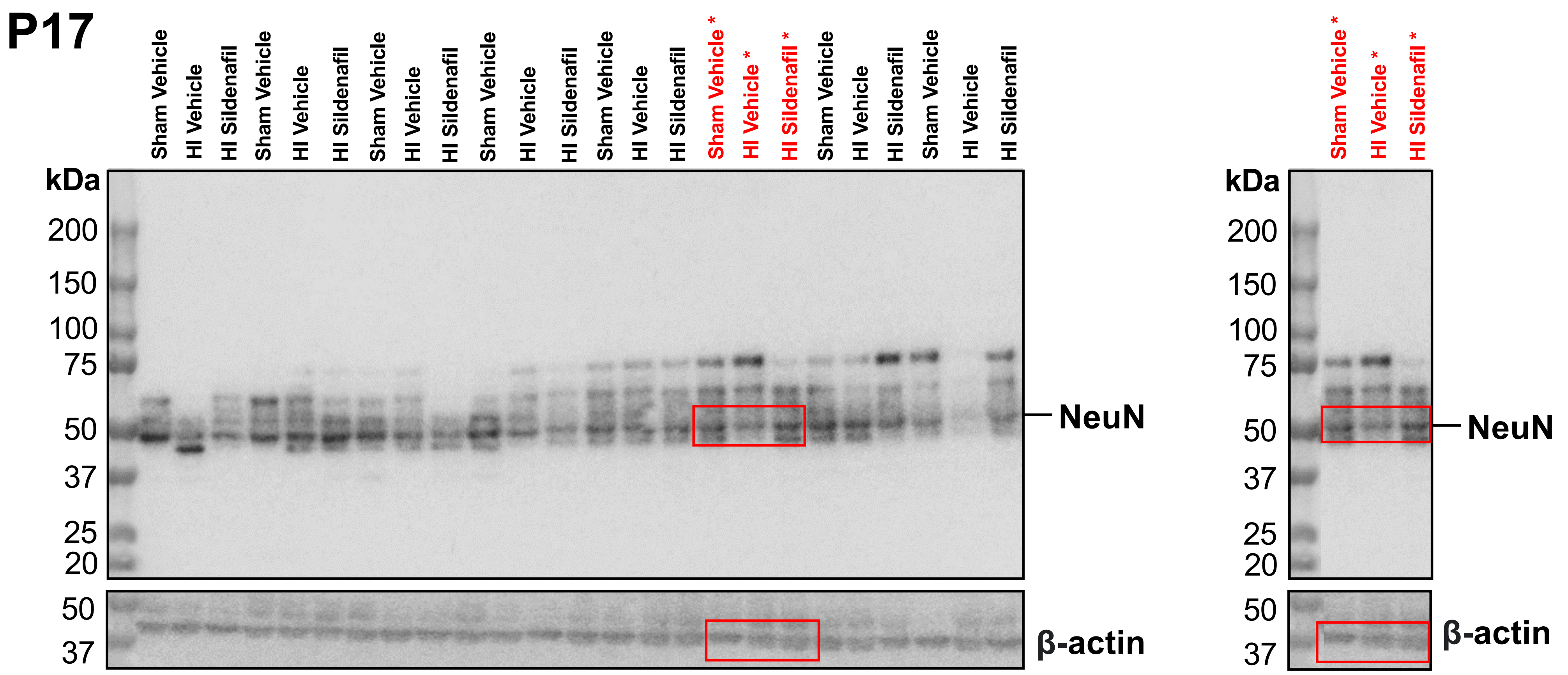


**FIGURE 3D – P17: Western blotting for NeuN at P17**, showing full-length western blots (on left panel) and representative samples chosen to be cropped for main Figure 3 (on right panel). The regions of the original blots used in main figure have been denoted using red boxes.

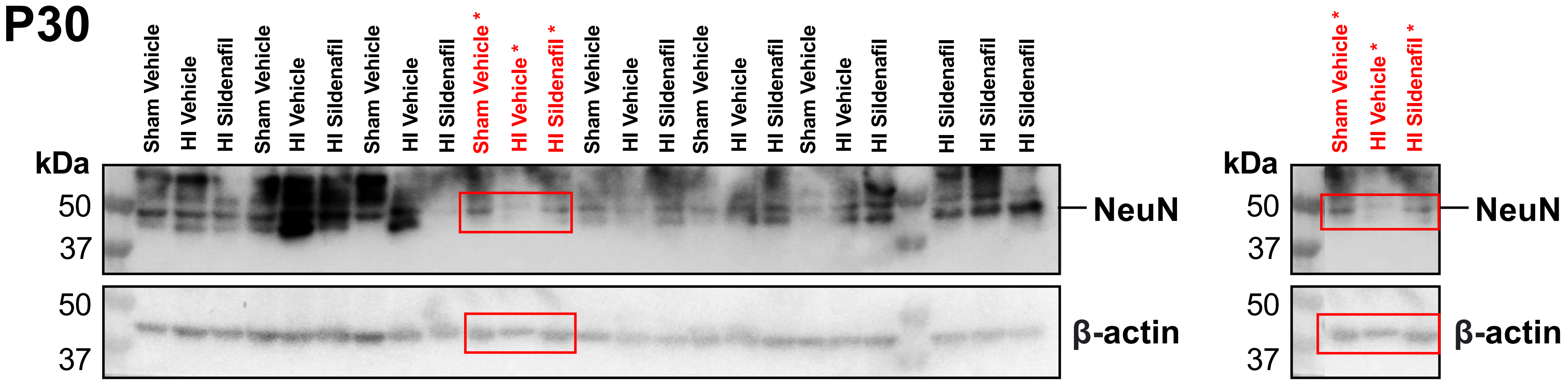


**FIGURE 3D – P30: Western blotting for NeuN at P30**, showing full-length western blots (on left panel) and representative samples chosen to be cropped for main Figure 3 (on right panel). The regions of the original blots used in main figure have been denoted using red boxes.

# Calretinin

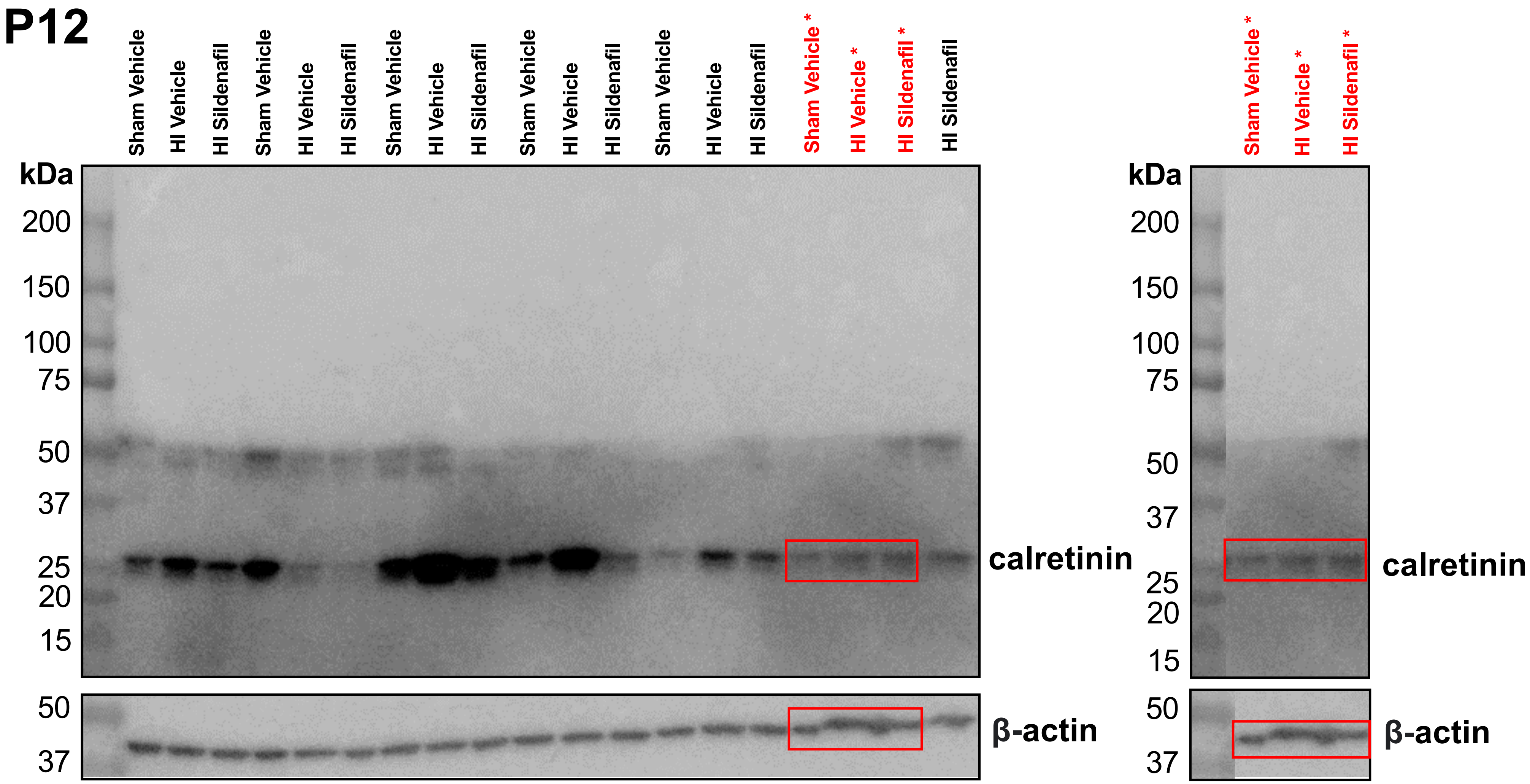


**FIGURE 3E – P12: Western blotting for Calretinin at P12**, showing full-length western blots (on left panel) and representative samples chosen to be cropped for main Figure 3 (on right panel). The regions of the original blots used in main figure have been denoted using red boxes.

P17

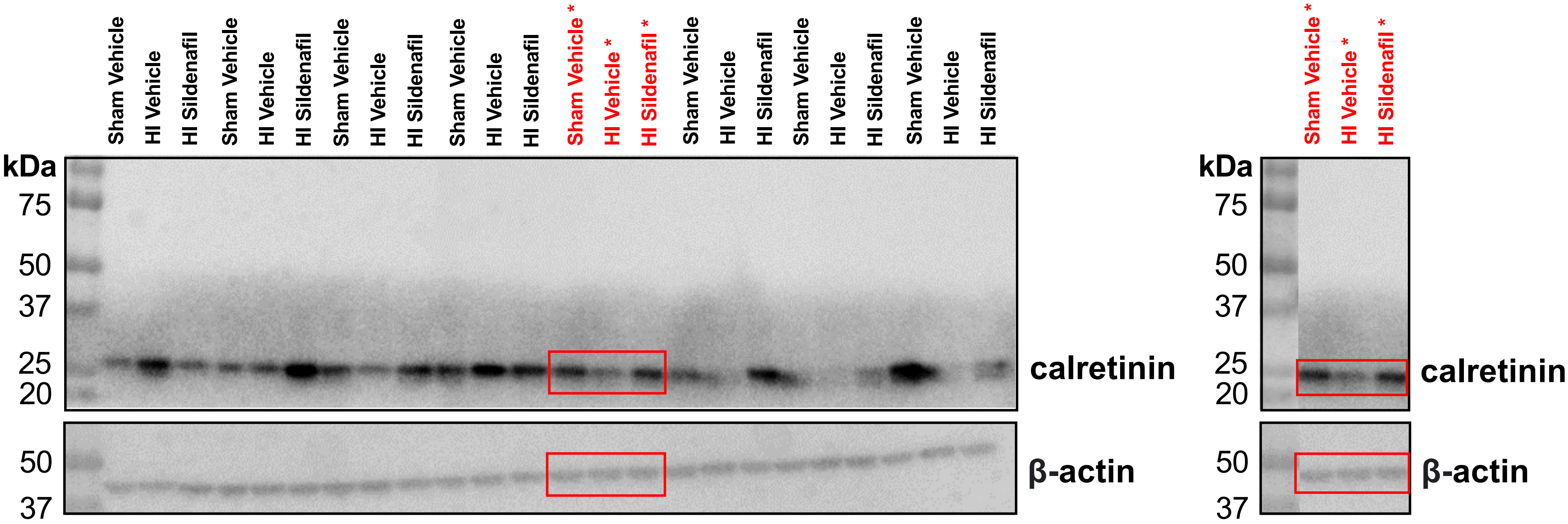


**FIGURE 3E – P17: Western blotting for Calretinin at P17**, showing full-length western blots (on left panel) and representative samples chosen to be cropped for main Figure 3 (on right panel). The regions of the original blots used in main figure have been denoted using red boxes.

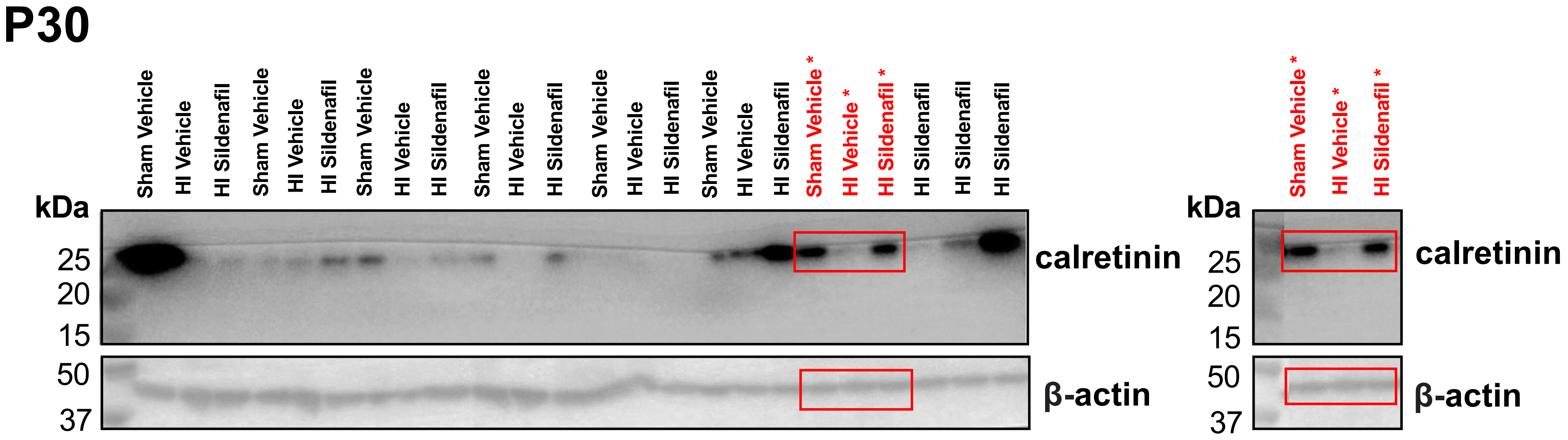


**FIGURE 3E – P30: Western blotting for Calretinin at P30**, showing full-length western blots (on left panel) and representative samples chosen to be cropped for main Figure 3 (on right panel). The regions of the original blots used in main figure have been denoted using red boxes.

# Calbindin

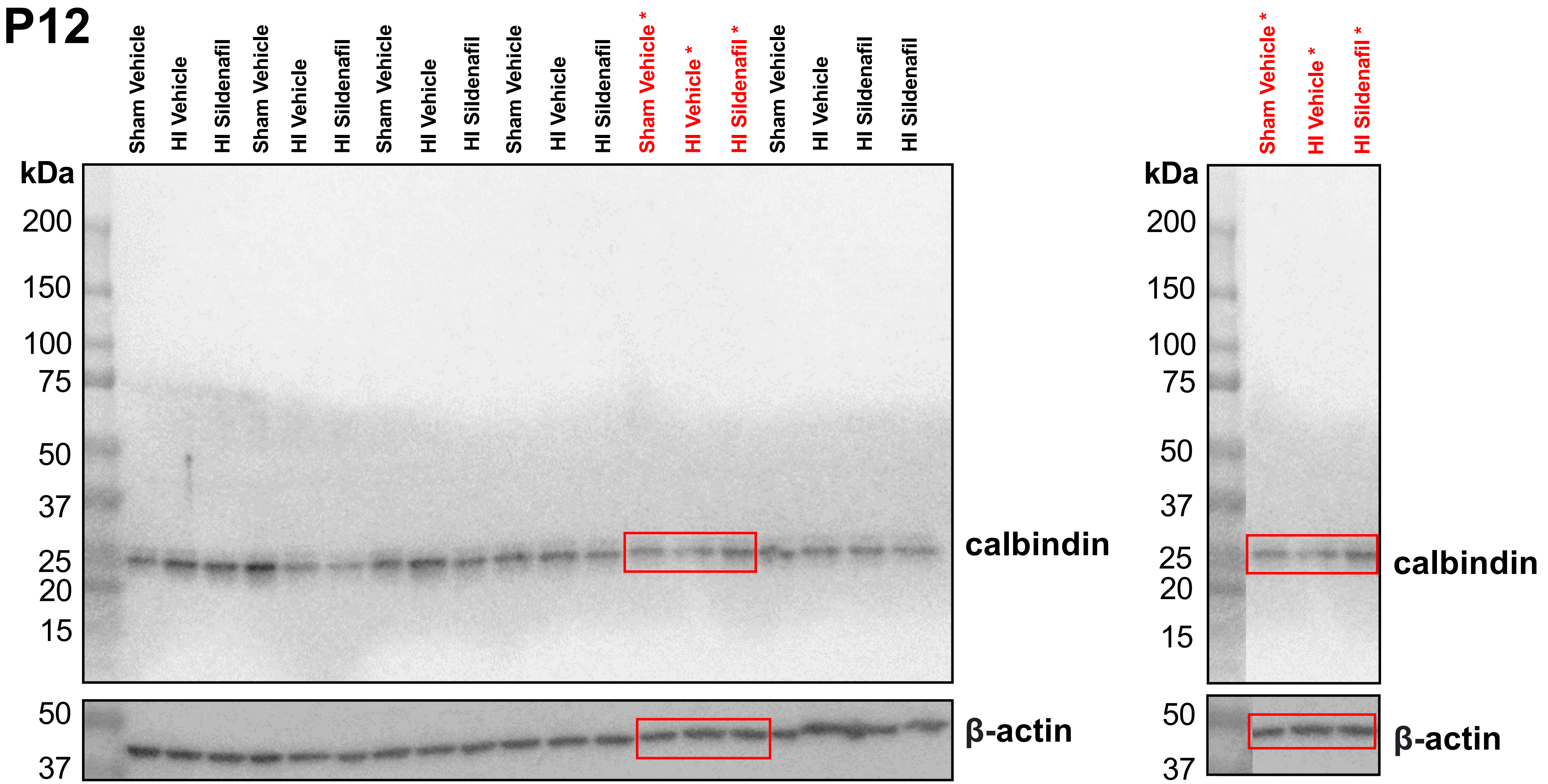


**FIGURE 3F – P12: Western blotting for Calbindin at P12**, showing full-length western blots (on left panel) and representative samples chosen to be cropped for main Figure 3 (on right panel). The regions of the original blots used in main figure have been denoted using red boxes.

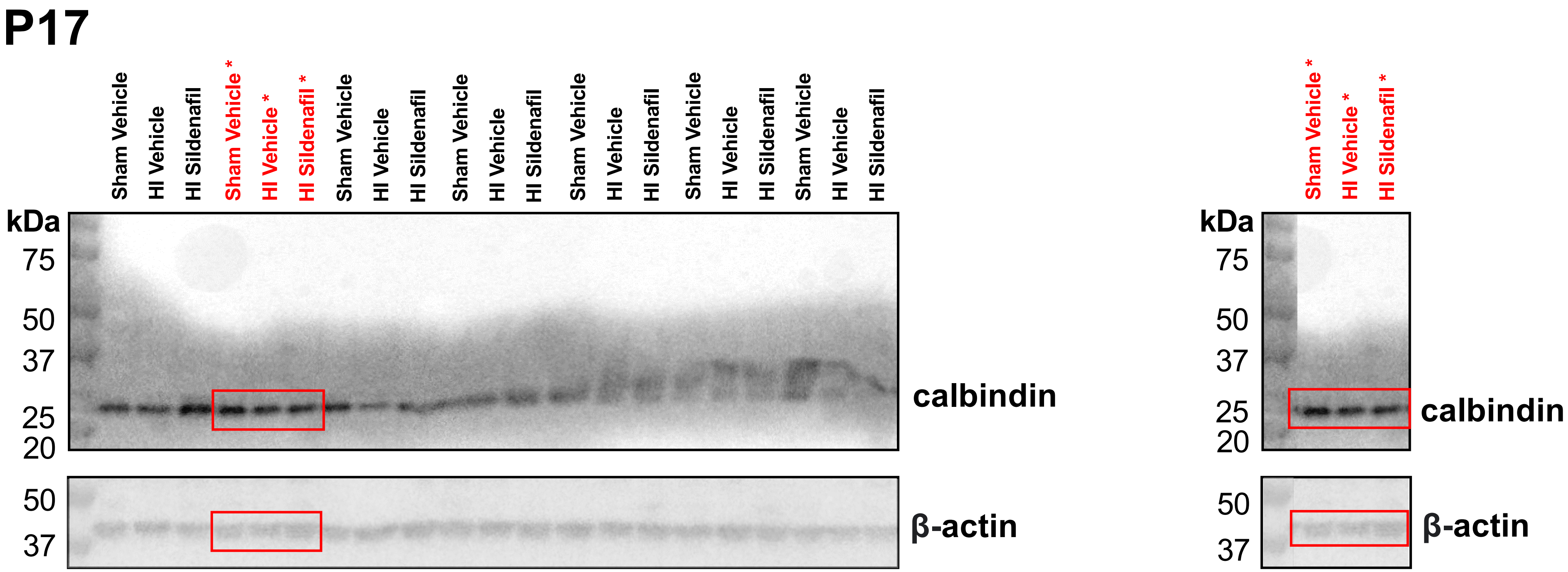


**FIGURE 3F – P17: Western blotting for Calbindin at P17**, showing full-length western blots (on left panel) and representative samples chosen to be cropped for main Figure 3 (on right panel). The regions of the original blots used in main figure have been denoted using red boxes.

# P30

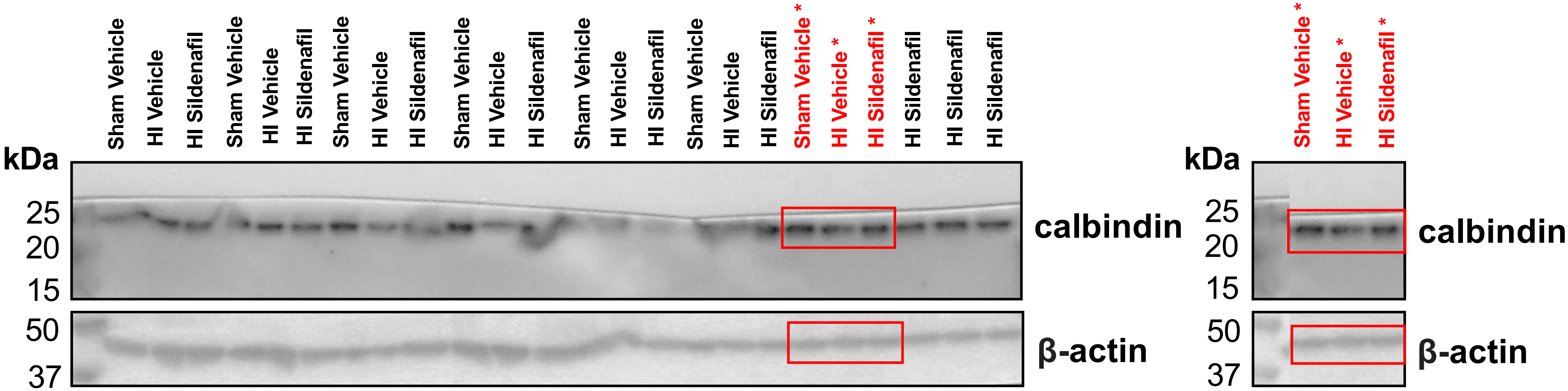


**FIGURE 3F – P30: Western blotting for Calbindin at P30**, showing full-length western blots (on left panel) and representative samples chosen to be cropped for main Figure 3 (on right panel). The regions of the original blots used in main figure have been denoted using red boxes.

# PARP

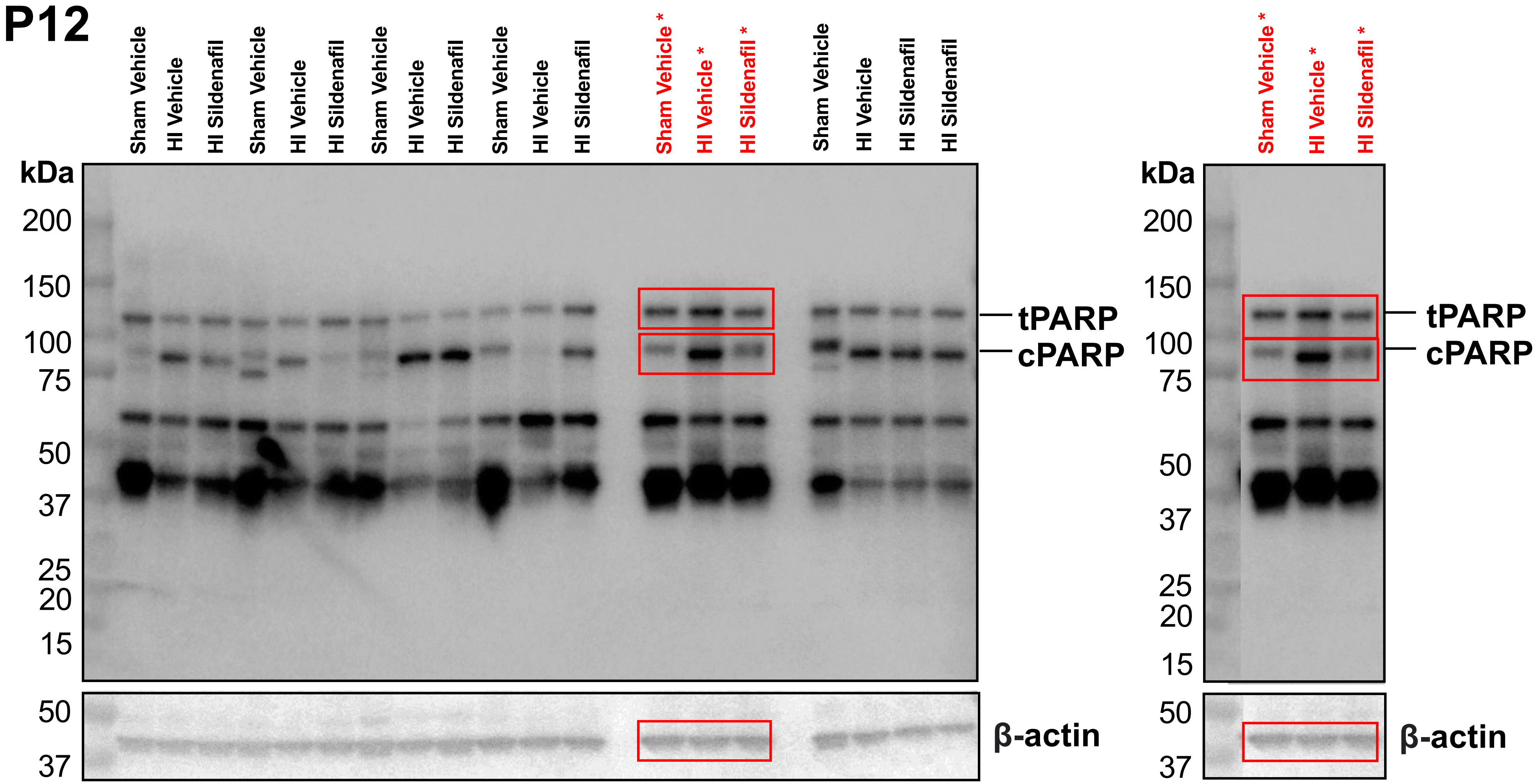


**FIGURE 3G – P12: Western blotting for PARP at P12**, showing full-length western blots (on left panel) and representative samples chosen to be cropped for main Figure 3 (on right panel). The regions of the original blots used in main figure have been denoted using red boxes.

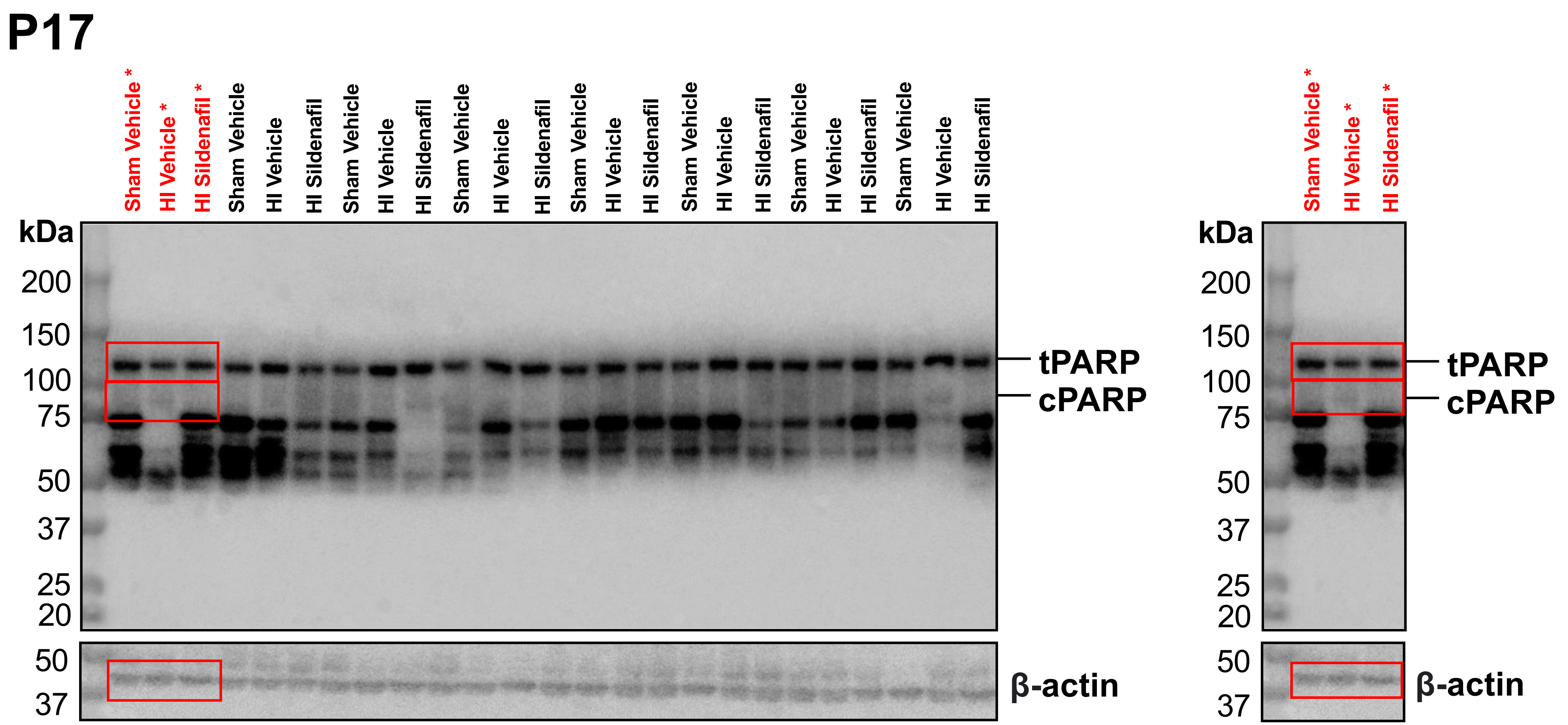


**FIGURE 3G – P17: Western blotting for PARP at P17**, showing full-length western blots (on left panel) and representative samples chosen to be cropped for main Figure 3 (on right panel). The regions of the original blots used in main figure have been denoted using red boxes.

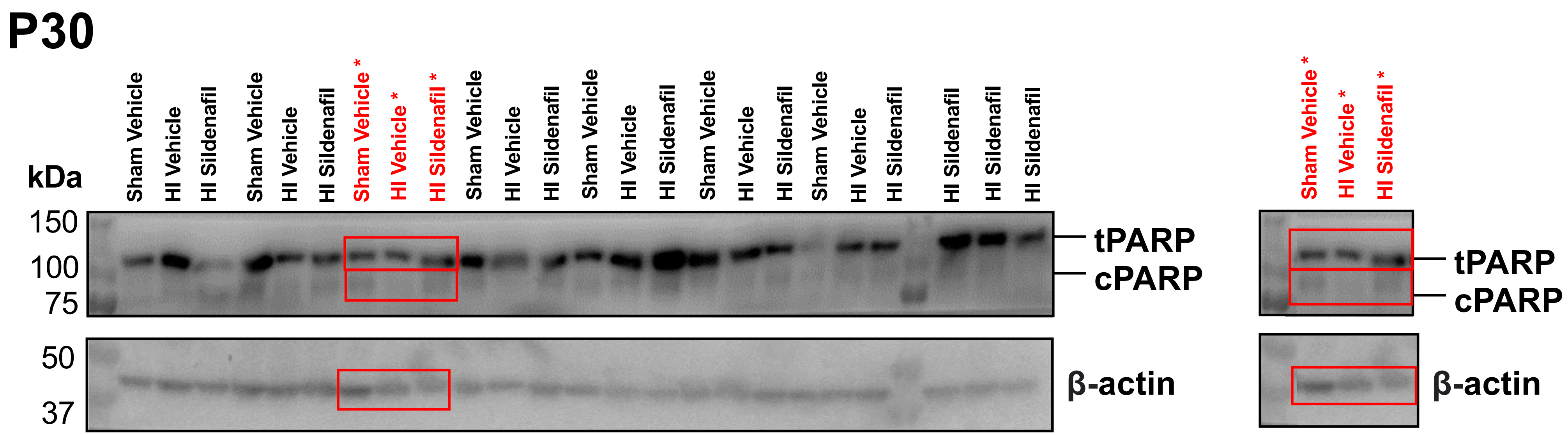


**FIGURE 3G – P30: Western blotting for PARP at P30**, showing full-length western blots (on left panel) and representative samples chosen to be cropped for main Figure 3 (on right panel). The regions of the original blots used in main figure have been denoted using red boxes.

# GAP43

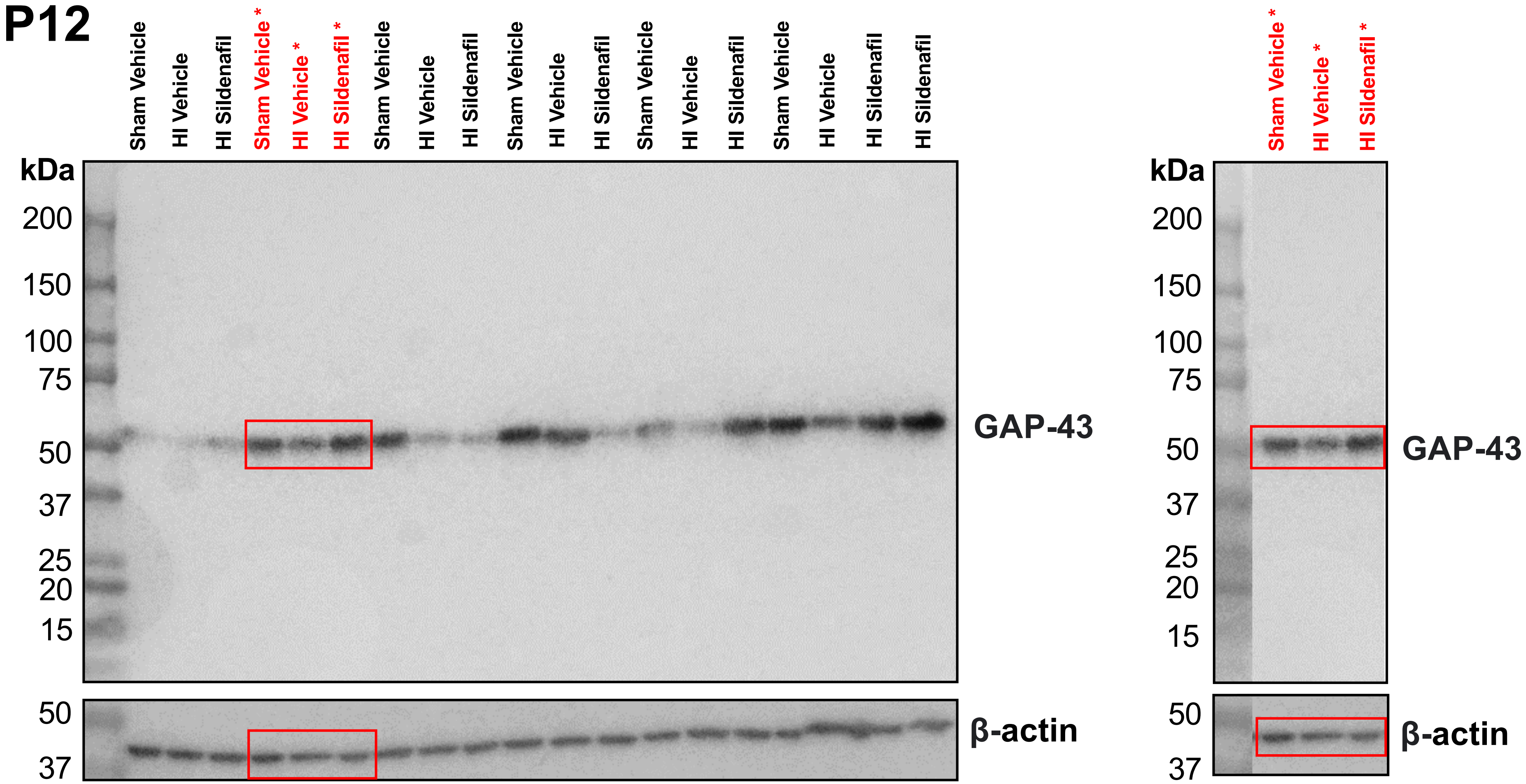


**FIGURE 3H – P12: Western blotting for GAP-43 at P12**, showing full-length western blots (on left panel) and representative samples chosen to be cropped for main Figure 3 (on right panel). The regions of the original blots used in main figure have been denoted using red boxes.

# P17

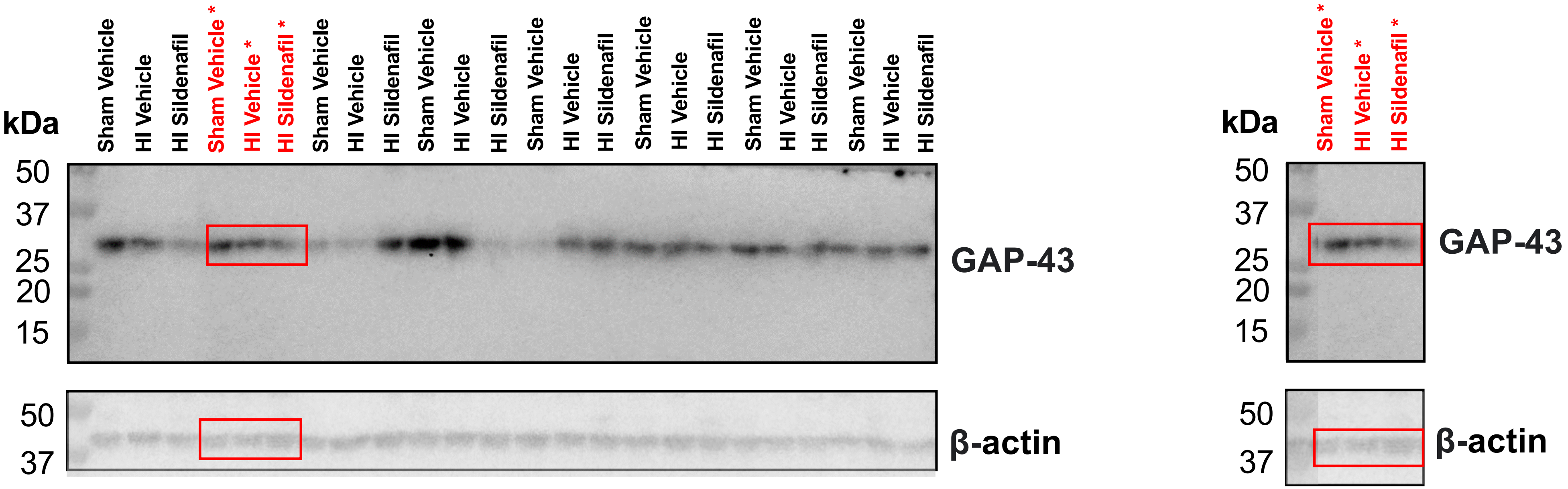


**FIGURE 3H – P17: Western blotting for GAP-43 at P17**, showing full-length western blots (on left panel) and representative samples chosen to be cropped for main Figure 3 (on right panel). The regions of the original blots used in main figure have been denoted using red boxes.

## P30

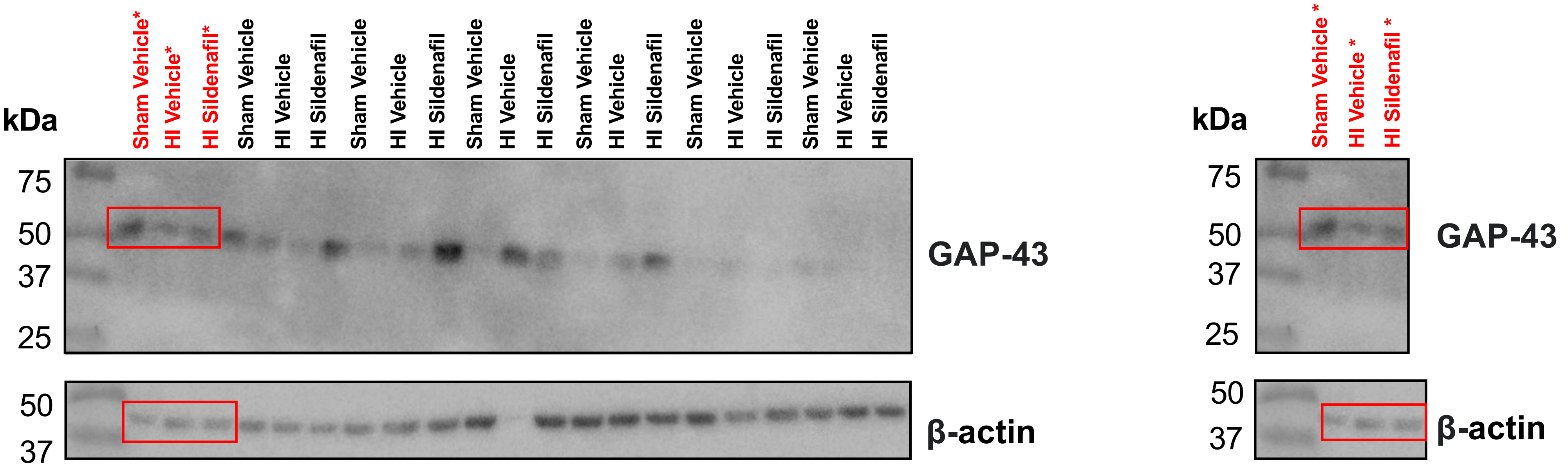


**FIGURE 3H – P30: Western blotting for GAP-43 at P30**, showing full-length western blots (on left panel) and representative samples chosen to be cropped for main Figure 3 (on right panel). The regions of the original blots used in main figure have been denoted using red boxes.

# pAKT

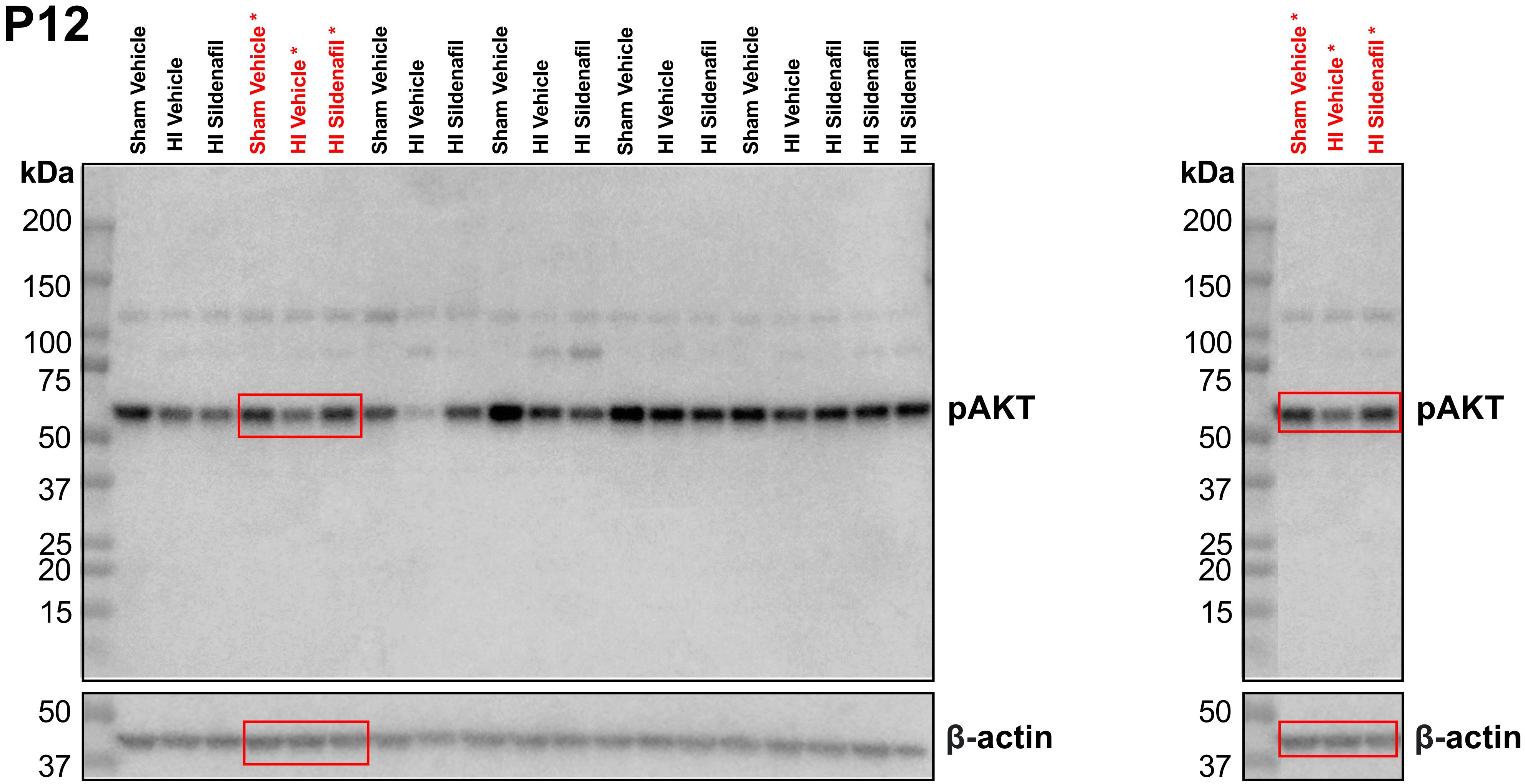


**FIGURE 6A – P12: Western blotting for pAKT at P12**, showing full-length western blots (on left panel) and representative samples chosen to be cropped for main Figure 3 (on right panel). The regions of the original blots used in main figure have been denoted using red boxes.

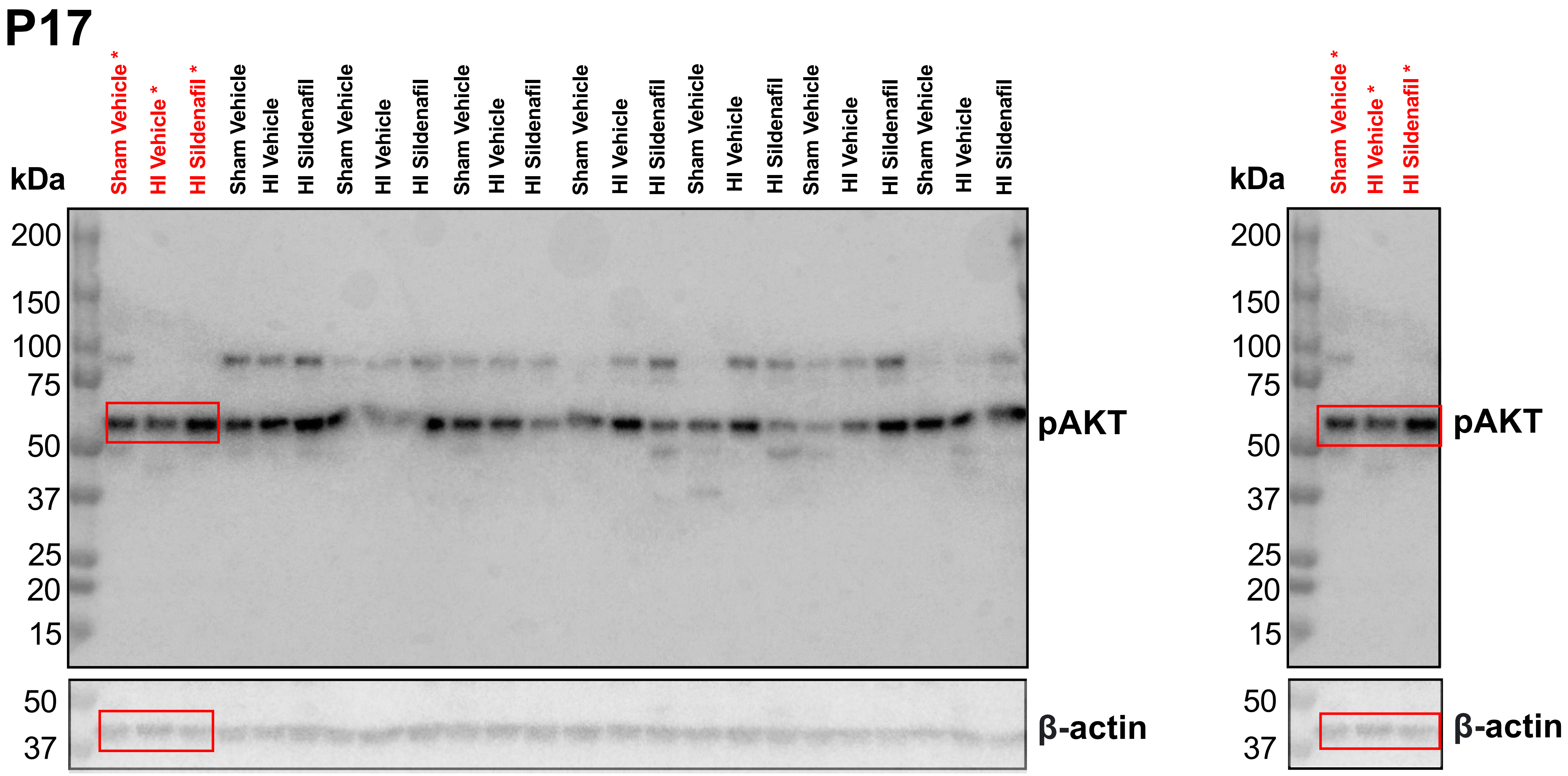


**FIGURE 6A – P17: Western blotting for pAKT at P17**, showing full-length western blots (on left panel) and representative samples chosen to be cropped for main Figure 3 (on right panel). The regions of the original blots used in main figure have been denoted using red boxes.

# P30

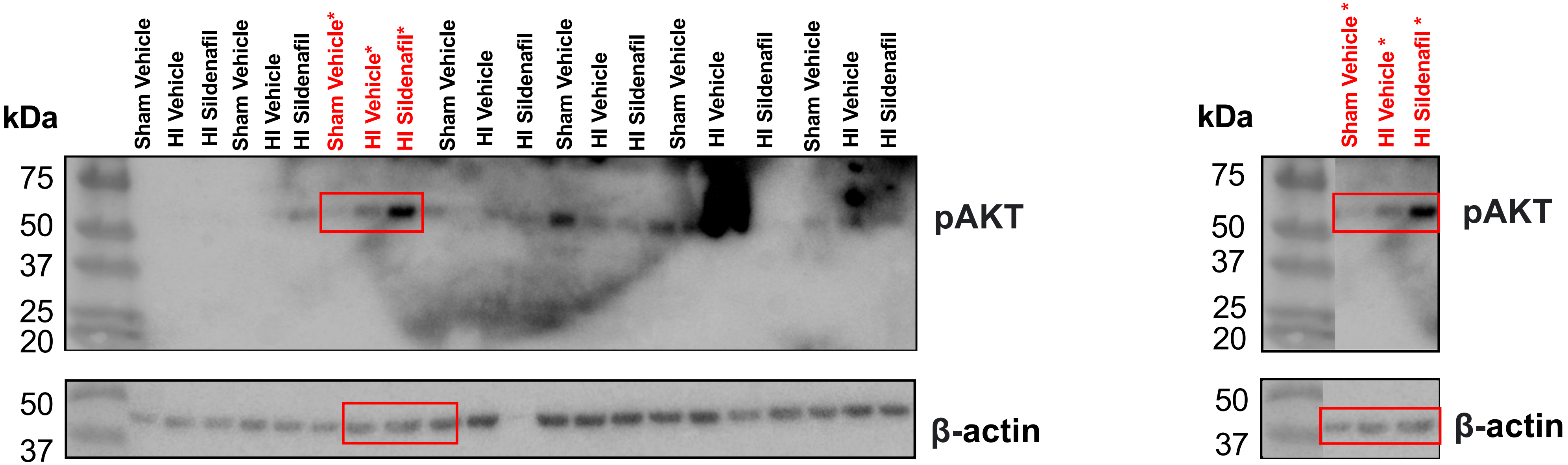


**FIGURE 6A – P30: Western blotting for pAKT at P30**, showing full-length western blots (on left panel) and representative samples chosen to be cropped for main Figure 3 (on right panel). The regions of the original blots used in main figure have been denoted using red boxes.

# mTOR2481

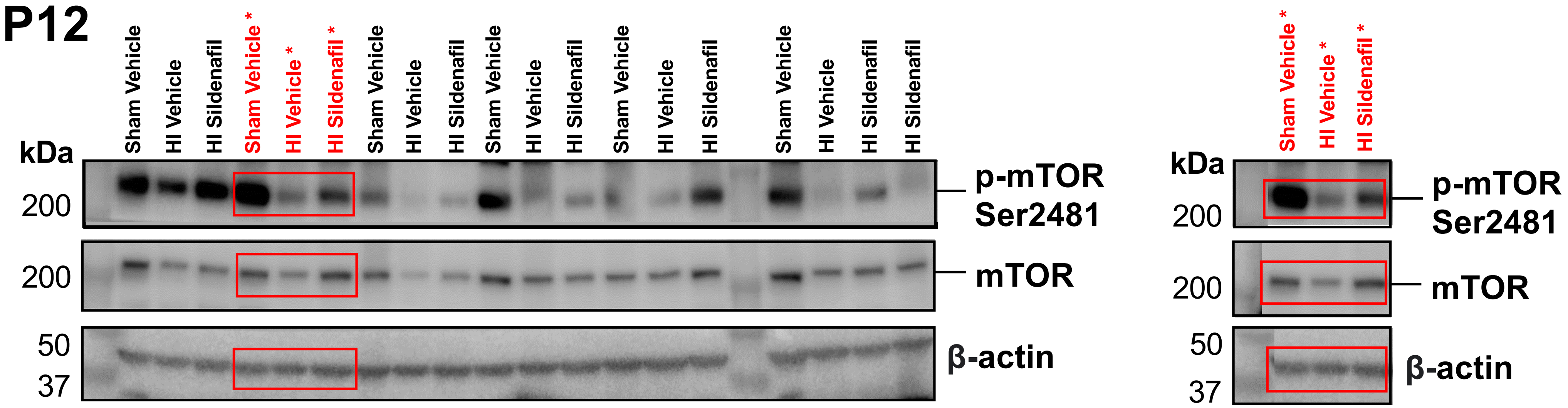


**FIGURE 6B – P12: Western blotting for mTOR Ser 2481 at P12**, showing full-length western blots (on left panel) and representative samples chosen to be cropped for main Figure 3 (on right panel). The regions of the original blots used in main figure have been denoted using red boxes.

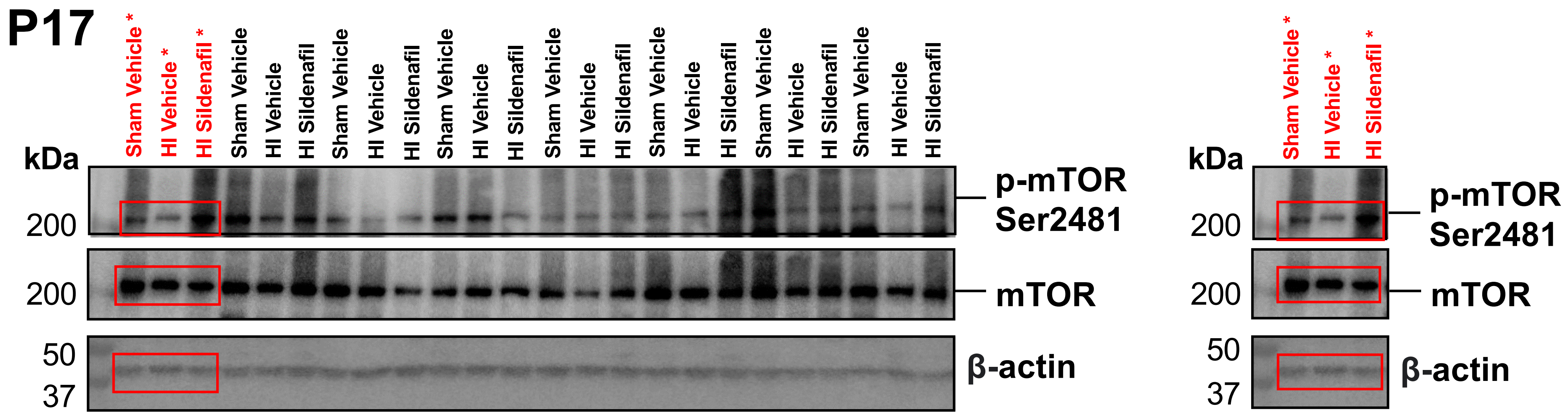


**FIGURE 6B – P17: Western blotting for mTOR Ser 2481 at P17**, showing full-length western blots (on left panel) and representative samples chosen to be cropped for main Figure 3 (on right panel). The regions of the original blots used in main figure have been denoted using red boxes.

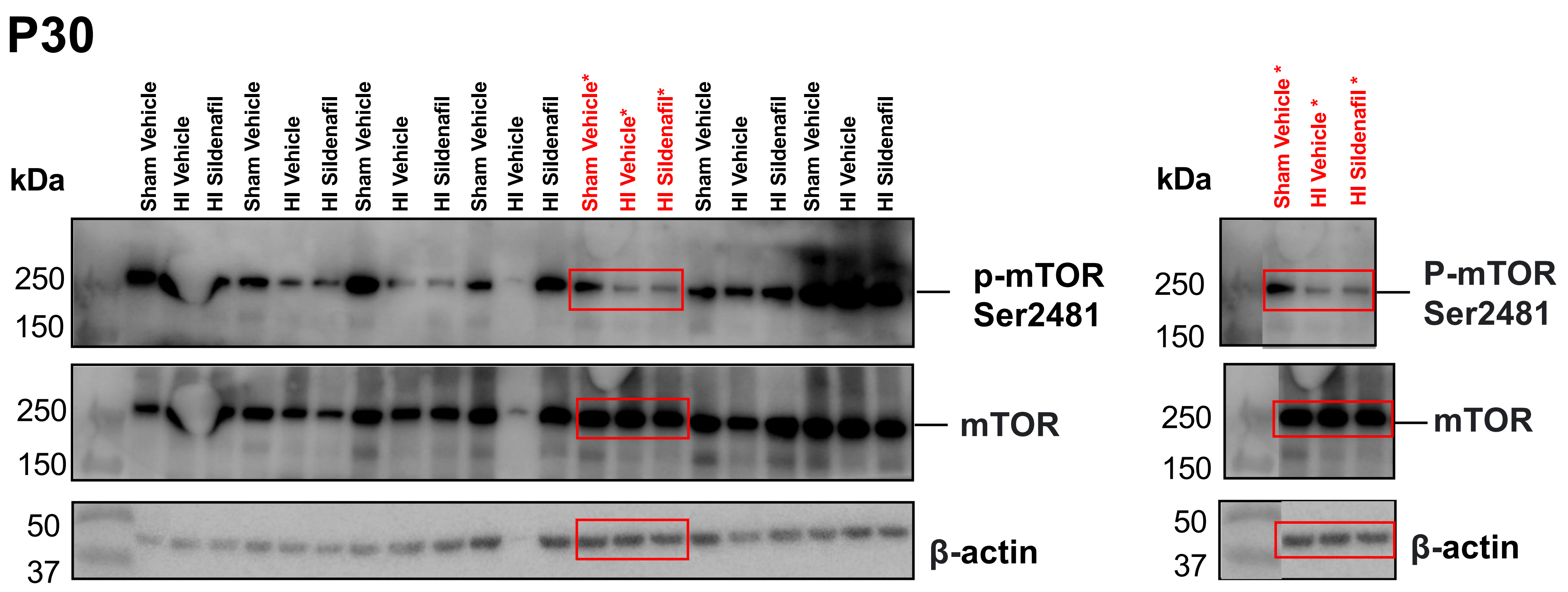


**FIGURE 6B – P30: Western blotting for mTOR Ser 2481 at P30**, showing full-length western blots (on left panel) and representative samples chosen to be cropped for main Figure 3 (on right panel). The regions of the original blots used in main figure have been denoted using red boxes.

# mTOR 2448

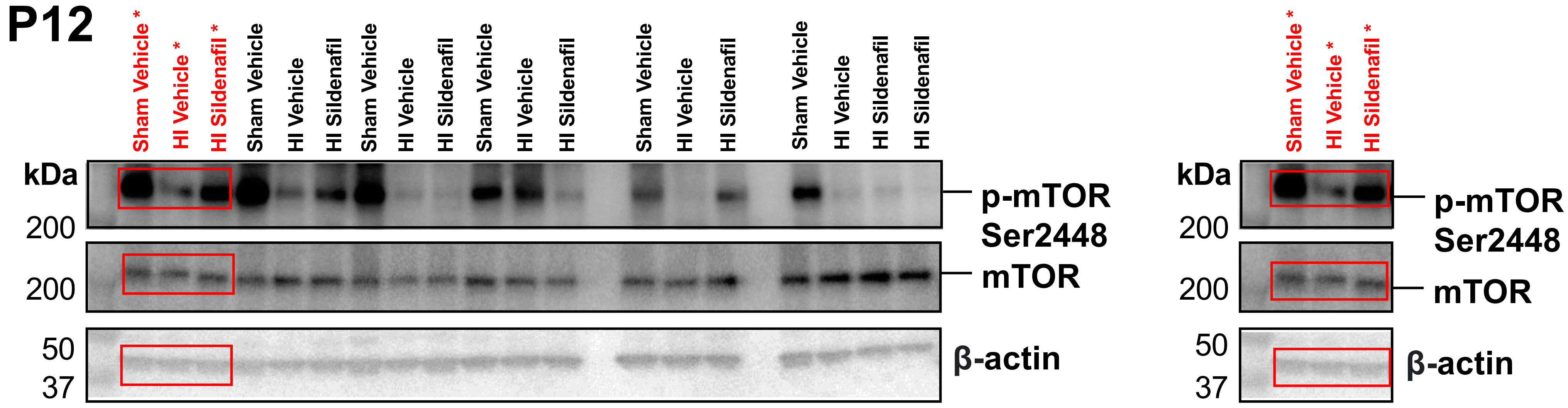


**FIGURE 6C – P12: Western blotting for mTOR Ser 2448 at P12**, showing full-length western blots (on left panel) and representative samples chosen to be cropped for main Figure 3 (on right panel). The regions of the original blots used in main figure have been denoted using red boxes.

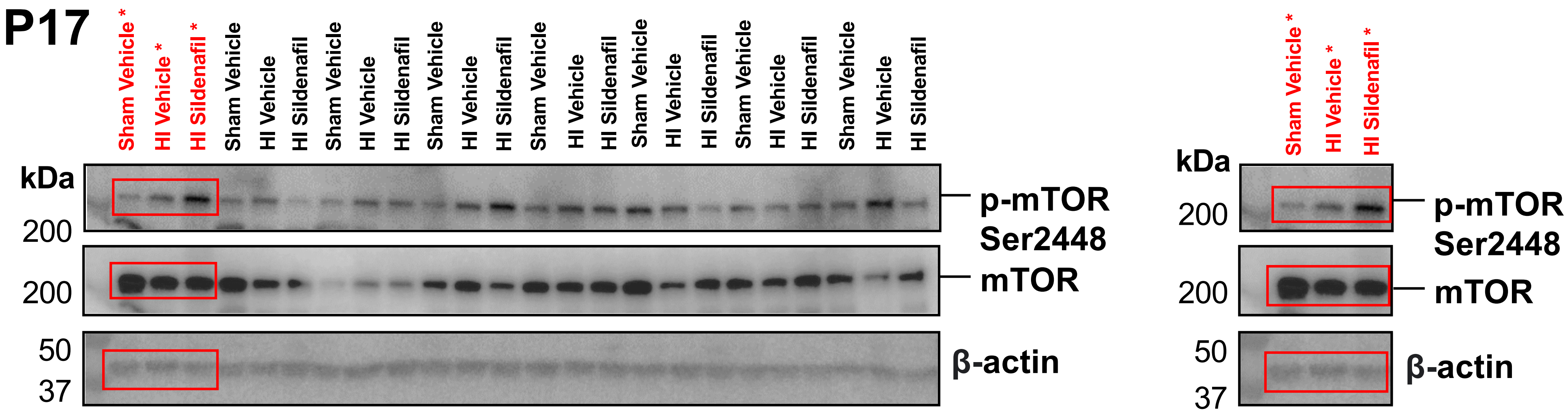


**FIGURE 6C – P17: Western blotting for mTOR Ser 2448 at P17**, showing full-length western blots (on left panel) and representative samples chosen to be cropped for main Figure 3 (on right panel). The regions of the original blots used in main figure have been denoted using red boxes.

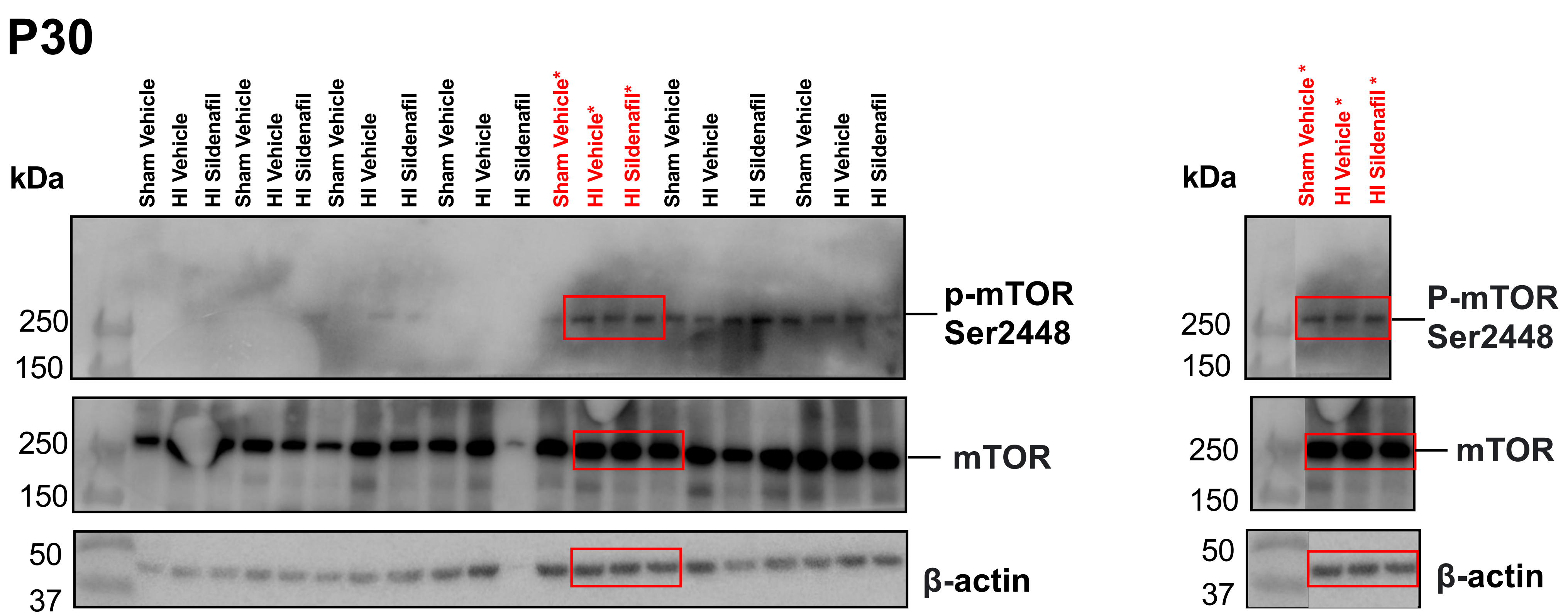


**FIGURE 6C – P30: Western blotting for mTOR Ser 2448 at P30**, showing full-length western blots (on left panel) and representative samples chosen to be cropped for main Figure 3 (on right panel). The regions of the original blots used in main figure have been denoted using red boxes.
